# Thermal, water, and land cover factors led to contrasting urban and rural vegetation resilience to extreme hot months

**DOI:** 10.1093/pnasnexus/pgae147

**Published:** 2024-04-15

**Authors:** Yaoping Wang, Jiafu Mao, Christa M Brelsford, Daniel M Ricciuto, Fengming Yuan, Xiaoying Shi, Deeksha Rastogi, Melanie M Mayes, Shih-Chieh Kao, Jeffrey M Warren, Natalie A Griffiths, Xinghua Cheng, David J Weston, Yuyu Zhou, Lianhong Gu, Peter E Thornton

**Affiliations:** Environmental Sciences Division and Climate Change Science Institute, Oak Ridge National Laboratory, Oak Ridge, TN 37830, USA; Environmental Sciences Division and Climate Change Science Institute, Oak Ridge National Laboratory, Oak Ridge, TN 37830, USA; Geospatial Science and Human Security Division, Oak Ridge National Laboratory, Oak Ridge, TN 37830, USA; Analytics, Intelligence and Technology Division, Los Alamos National Laboratory, Los Alamos, NM 87545, USA; Environmental Sciences Division and Climate Change Science Institute, Oak Ridge National Laboratory, Oak Ridge, TN 37830, USA; Environmental Sciences Division and Climate Change Science Institute, Oak Ridge National Laboratory, Oak Ridge, TN 37830, USA; Environmental Sciences Division and Climate Change Science Institute, Oak Ridge National Laboratory, Oak Ridge, TN 37830, USA; Computational Science and Engineering Division, Oak Ridge National Laboratory, Oak Ridge, TN 37830, USA; Environmental Sciences Division and Climate Change Science Institute, Oak Ridge National Laboratory, Oak Ridge, TN 37830, USA; Environmental Sciences Division and Climate Change Science Institute, Oak Ridge National Laboratory, Oak Ridge, TN 37830, USA; Environmental Sciences Division and Climate Change Science Institute, Oak Ridge National Laboratory, Oak Ridge, TN 37830, USA; Environmental Sciences Division and Climate Change Science Institute, Oak Ridge National Laboratory, Oak Ridge, TN 37830, USA; Department of Natural Resources and the Environment, University of Connecticut, Storrs, CT 06269, USA; Biosciences Division, Oak Ridge National Laboratory, Oak Ridge, TN 37830, USA; Department of Geography, The University of Hong Kong, Hong Kong, 999077, China; Environmental Sciences Division and Climate Change Science Institute, Oak Ridge National Laboratory, Oak Ridge, TN 37830, USA; Environmental Sciences Division and Climate Change Science Institute, Oak Ridge National Laboratory, Oak Ridge, TN 37830, USA

## Abstract

With continuing global warming and urbanization, it is increasingly important to understand the resilience of urban vegetation to extreme high temperatures, but few studies have examined urban vegetation at large scale or both concurrent and delayed responses. In this study, we performed an urban–rural comparison using the Enhanced Vegetation Index and months that exceed the historical 90th percentile in mean temperature (referred to as “hot months”) across 85 major cities in the contiguous United States. We found that hot months initially enhanced vegetation greenness but could cause a decline afterwards, especially for persistent (≥4 months) and intense (≥+2 °C) episodes in summer. The urban responses were more positive than rural in the western United States or in winter, but more negative during spring–autumn in the eastern United States. The east–west difference can be attributed to the higher optimal growth temperatures and lower water stress levels of the western urban vegetation than the rural. The urban responses also had smaller magnitudes than the rural responses, especially in deciduous forest biomes, and least in evergreen forest biomes. Within each biome, analysis at 1 km pixel level showed that impervious fraction and vegetation cover, local urban heat island intensity, and water stress were the key drivers of urban–rural differences. These findings advance our understanding of how prolonged exposure to warm extremes, particularly within urban environments, affects vegetation greenness and vitality. Urban planners and ecosystem managers should prioritize the long and intense events and the key drivers in fostering urban vegetation resilience to heat waves.

**Significance Statement**

Understanding urban vegetation’s resilience to hot extremes is vital for urban planning and well-being amid global warming and urbanization, yet it is understudied on a large scale. Analyzing satellite data from 85 large US cities, this study reveals: (i) prolonged (≥4 months) and intense (≥+2 °C) summer heat extremes cause a delayed but general decline in vegetation greenness after an initial increase, (ii) urban vegetation shows more resilience in the arid west but less in the humid east, explainable by optimal growth temperature and moisture sensitivity differences, and (iii) within smaller areas, the disparity in urban–rural vegetation resilience is mainly influenced by impervious surfaces, vegetation cover, local urban heat island intensity, and moisture sensitivity.

## Introduction

Ecosystem resilience to disturbances (e.g. extreme climate events) is a topic of longstanding interest to ecologists, and the literature contains two branches: ecological resilience and engineering resilience. Ecological resilience examines the ability of an ecosystem to absorb large disturbances without shifting to a different domain of attraction in structure or functioning, while engineering resilience examines the departure and return of an ecosystem to its reference state after small disturbances (1–3). Engineering resilience is often employed to quantify the short-term responses of vegetation to climate extremes (4–7), and the concept requires quantifying both the concurrent vegetation response, i.e. during the disturbance, and the delayed response, i.e. after the disturbance (2, 8). Many metrics have been established for both types of responses. Metrics for the concurrent response are usually called “resistance,” while metrics for the delayed response are called “resilience,” “recovery,” or variations of those (e.g. rate of recovery) (2, 8).

With global warming leading to more frequent and intense high temperatures, a better understanding of vegetation resilience to such conditions is of scientific and practical importance (9). Excessive heat can cause physiological damage and increase the susceptibility of vegetation to other stressors (10–12), but warm extremes below the optimal temperature of vegetation growth may have positive effects (13). Large-scale studies over areas dominated by natural vegetation and crops have shown that whether vegetation responds positively or negatively to warm extremes depends on prevailing temperature, moisture, and land cover conditions (13–19). However, those studies generally focused on concurrent responses and did not use the resilience framework to examine delayed responses (13–18). Studies on a few prominent continental-scale, multimonth extremely warm periods showed that spring heat waves had delayed negative impacts on vegetation growth later in the summer (19, 20). Yet it remains to be examined whether delayed negative vegetation responses occur during less prominent events and all seasons.

Past studies on vegetation responses to warm extremes typically focused on large areas dominated by natural vegetation and crops, with only one study including urban and built-up areas as a covariate (15). There is an urgent need to better understand the resilience of urban vegetation to warm extremes for several reasons. Urban vegetation provides ecosystem services such as mitigating the urban heat island, improving air quality, sequestering carbon, providing habitats, and providing recreational value (21). Many US cities are planting trees as a means to fight climate change (22), and understanding the resilience of urban vegetation to warm extremes will provide valuable information for cost–benefit analysis of such projects. Some characteristics of the urban environment, such as the urban heat island, higher CO_2_ and air pollutant concentrations, higher nitrogen deposition, and higher human disturbance, are similar to expected changes in natural ecosystems under intensifying human activities (23–25). Therefore, many consider urban environments to be a natural laboratory for studying possible ecosystem responses to future global environmental change (23, 26). The urban environment also has distinct characteristics, such as irrigation of outdoor landscapes (27, 28). The unique urban environment gives rise to many differences between urban and rural vegetation, such as earlier spring leaf-out, later autumn senescence, and higher greenness per unit vegetated area in urban vegetation than rural vegetation (29–32). However, even among case studies on individual cities, the resilience of urban vegetation has only been assessed with respect to drought, construction, and coinciding drought and heat waves (33, 34).

In this study, we contribute to existing literature by using the resilience framework to quantify both concurrent and delayed responses of vegetation greenness to months that exceed the historical 90th percentile in mean temperature (referred to as “hot months”). We ask (i) whether the urban and rural resilience are significantly different and (ii) if different, what environmental drivers are behind the differences across spatial scales. The spatial scope of the study was 85 major cities that are large enough to exhibit strong urban heat island effects, covering all major climate zones and biomes across the contiguous United States (SI Section 1.1.4). The study period was 2001–2019. The two resilience metrics we used were (i) resistance, which compares the maximum change in monthly Enhanced Vegetation Index (EVI) during the hot months to the preevent EVI to reveal the concurrent response and (ii) recovery, which compares the average EVI during the 3 months after the hot months to the preevent EVI to reveal the delayed response (SI Section 1.2.2; Fig. S1). That is, the two metrics are large and positive when the EVI increases above the preevent level, large and negative when the EVI declines below the preevent level, and near zero when the EVI remains similar to the preevent level. In the rest of the paper, we use “resilience,” “resilience metrics,” or “responses” when there is no need to distinguish between concurrent and delayed responses, and use “resistance” or “recovery” when we refer to either metric individually. To prevent confounding influences from natural seasonality and phenological changes, we deseasonalized the EVI prior to all calculations and defined the hot months using month-specific thresholds (SI Section 1.2.1). More specifically, a month is “hot” if its monthly mean temperature exceeds the historical 90th percentile threshold during the same month of the year. Consecutive months passing the threshold were merged into one “hot months” event (SI Section 1.2.1). The resulting events generally lasted between 1 and 12 months.

We found that in both urban and rural areas, hot months generally led to positive resistances, but long and intense (≥ 4 months, ≥2 °C in average deviation from normal conditions) hot months in summer caused negative recoveries. The urban–rural differences exhibited similar patterns in both metrics. Positive resilience occurred significantly more frequently in the urban areas than the rural areas in winter over the contiguous United States and in the other seasons in the western United States, but less frequently in the other seasons in the eastern United States. The magnitudes of the resilience metrics were significantly lower in the urban than the rural areas in all the seasons and most of the cities. Using regression analysis, we further found that at the scale of the whole contiguous United States, the key controls on the urban–rural differences were the water stress status and optimal temperatures of the vegetation, the climatological temperature and average impervious area of the city, and the intensity of the hot months. At smaller spatial scales, pixel-to-pixel variations in impervious area and rural vegetation fraction, local urban heat island intensity, and water stress status of the vegetation were the key controls.

## Results

### Urban, rural, and urban–rural differences in resilience metrics

**Fig. 1.**
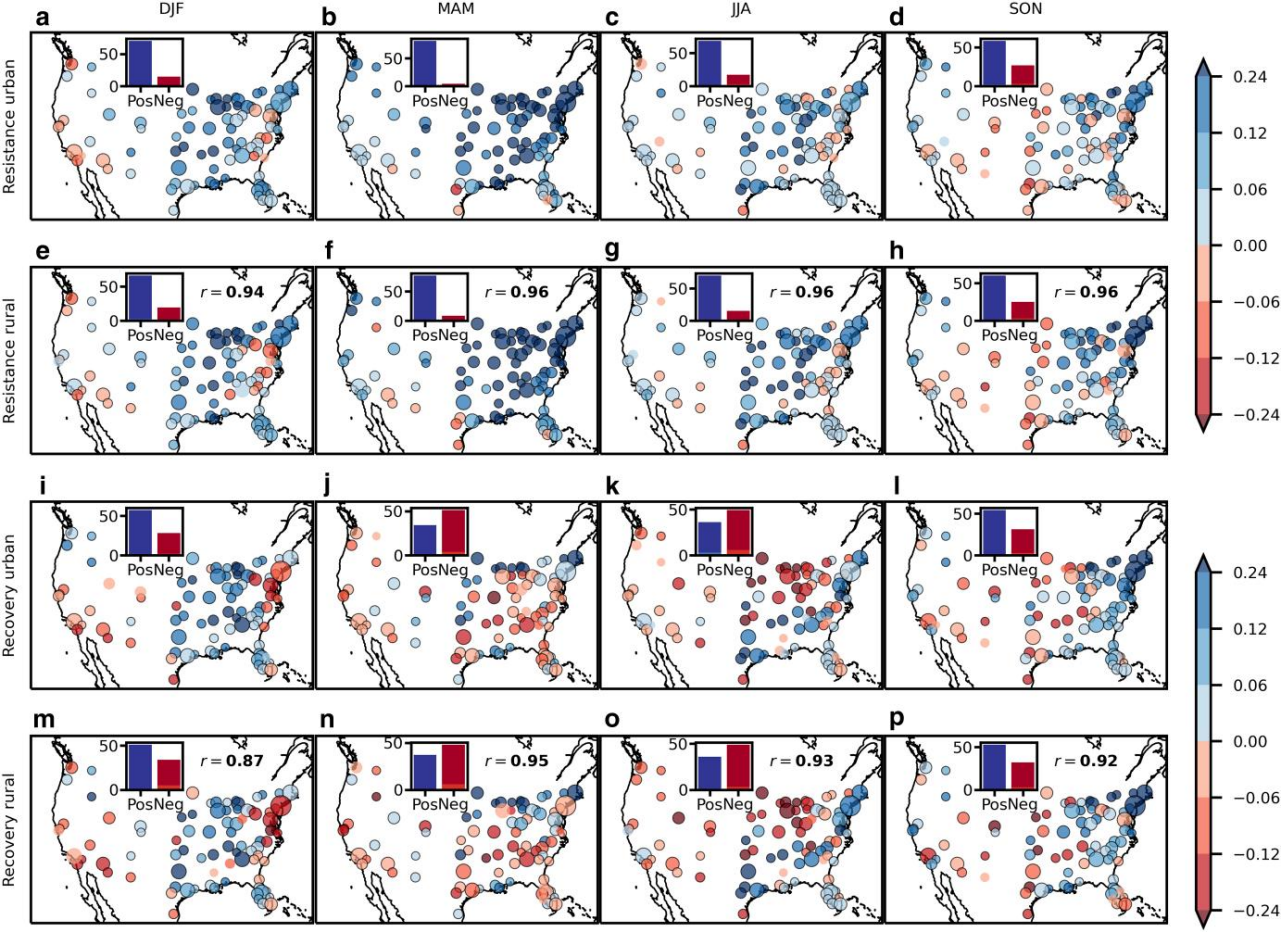
Urban median resistances a–d), recoveries e–h), rural median resistances i–l, and recoveries m–p) in each city. Season abbreviations: DJF—December to February, MAM—March to May, JJA—June to August, SON—September to November. The dot sizes are proportional to the square root of city sizes. Dots with edges indicate that the values were significantly different from zero at *P* ≤ 0.05, and dots without edges indicate insignificance. The inset bars show the number of cities with positive (Pos) and negative (Neg) median values, with the darker portions of the bars corresponding to the number of significant positive/negative dots, and the ligher portions insignificant dots. The *r* values in the rural panels are Spearman correlations between the urban and rural medians, with bold text meaning the values were significantly different from zero at *P* ≤ 0.05. Exact details on the calculation of all quantities are in SI Section 1.2.3.

In both the urban and rural areas, the EVI responses during the hot months, i.e. the resistances, were nearly always positive (Fig. 1a–h). The largest positive resistances occurred in spring (Fig. 1a–h). Also, the humid eastern United States saw more positive resistances than the arid western United States (Fig. 1a–h). During the 3 months after the hot months, the EVI showed mainly positive delayed responses, i.e. positive recoveries, in winter and autumn, but mainly negative delayed responses in spring and summer (Fig. 1i–p). The most prevalent negative recoveries occurred in spring, while the greatest magnitude of negative recoveries occurred in summer in the midwestern United States (Fig. 1i–p). Both the eastern and western United States experienced a mixture of positive and negative recoveries, but the recoveries in the eastern United States tended to have greater magnitudes (Fig. 1i–p). The urban responses were well correlated with their rural counterparts (Spearman correlations ≥ 0.87, Fig. 1).

**Fig. 2.**
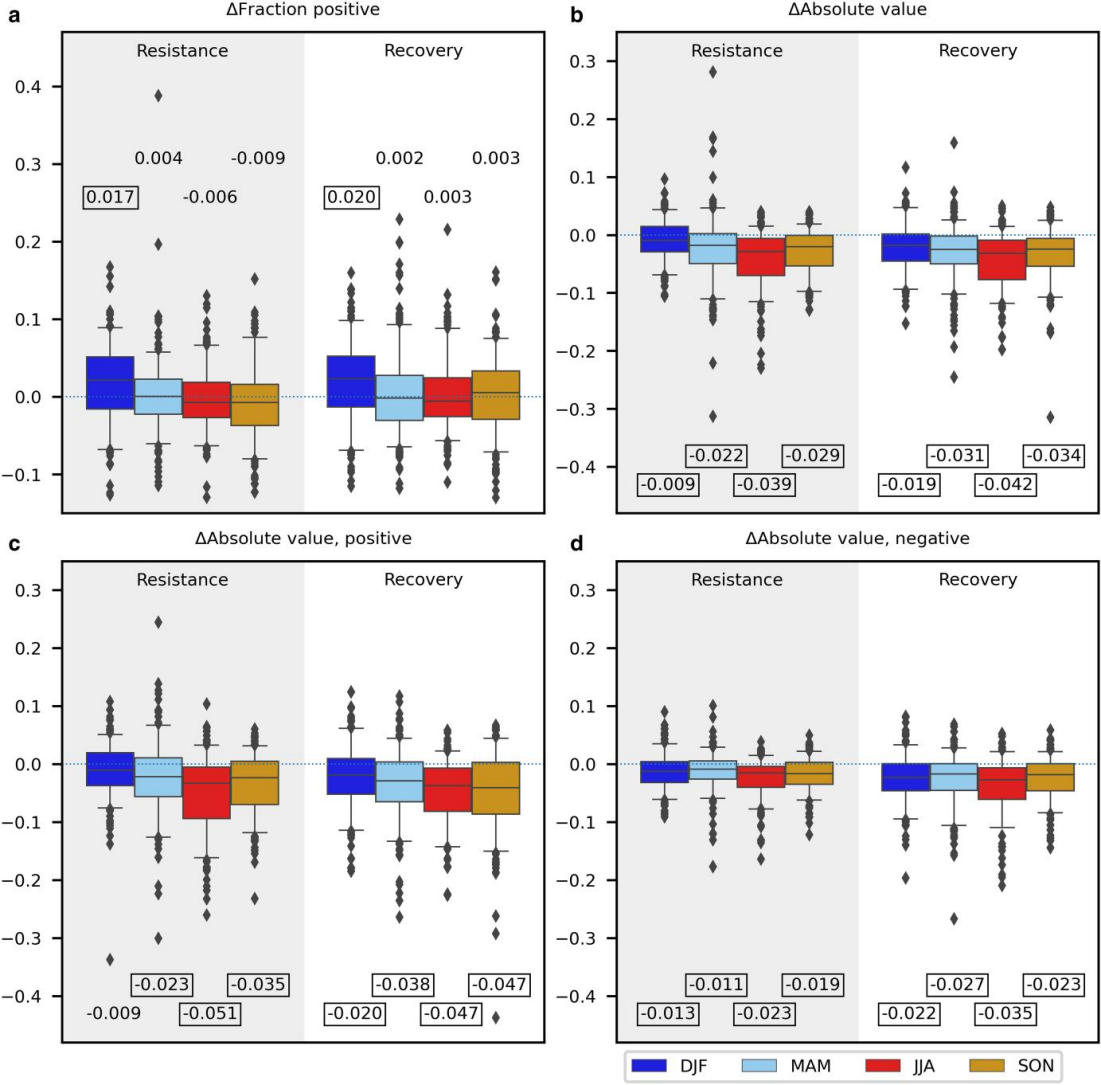
Distributions of urban–rural differences in the a) fraction of pixels with positive resistances and recoveries, b) median absolute resistances and recoveries over the pixels, c) median absolute resistances and recoveries over the pixels that had positive values, and d) median absolute resistances and recoveries over the pixels that had negative values, in each season. The partially shaded background in each subplot is used to distinguish the resistance boxplots from the recovery boxplots. The boxplots show, from top to bottom, the 95th, 75th, 50th, 25th, and 5th percentiles over all the hot months, cities, and meteorological datasets, and the fliers are values beyond the 5–95th percentile ranges. The number above or below each boxplot shows the value of the 50th percentile and whether it is significantly different from zero (bound by rectangle) at *P* ≤ 0.05. Season abbreviations: DJF—December to February, MAM—March to May, JJA—June to August, SON—September to November. Exact details on the calculation of all quantities are in SI Section 1.2.3.

Given that the number of positive responses typically exceeded the number of negative responses (Fig. 1), direct urban–rural comparison on the raw metrics can lead to potential confusion. For instance, a statistical test could suggest urban areas have systematically lower responses, either when the urban areas have a lower fraction of positive responses, or when the urban and rural areas have the same fractions but the urban responses’ magnitudes were always smaller. To prevent this confusion, we separately compared the signs and magnitudes of the responses. The comparison results, aggregated over all 85 cities, are shown in Fig. 2. The urban areas had a significantly higher fraction of positive responses than the rural areas in winter, but similar fractions in the other seasons (Fig, 2a). The magnitudes of the urban responses, on the other hand, were significantly lower than the rural responses in all the seasons, no matter whether the responses were positive or negative (Fig. 2b–d).

We also examined the spatial distributions of the urban–rural differences and found an east–west contrast in sign. In spring, summer, and autumn in the eastern cities, the urban areas had a higher fraction of positive responses than the rural areas (except for recovery in autumn), with statistical significance in summer (Table S3). In those same seasons in the western cities, the urban areas rather had a higher fraction of negative responses, with statistical significance in summer (Table S3). Therefore, the overall lack of statistical significance in those seasons over the contiguous United States was partially caused by cancellation (Fig. 2a). For urban–rural differences in magnitudes, there was no east–west contrast (Table S3). At the level of individual cities, the urban–rural differences in signs and magnitudes were both mostly statistically significant (Fig. S3i–p). The urban–rural differences in resistance and the urban–rural differences in recovery had similar seasonal and spatial patterns (Figs. 2 and S3a–h).

### Large-scale controls of urban–rural differences in resilience metrics

**Fig. 3.**
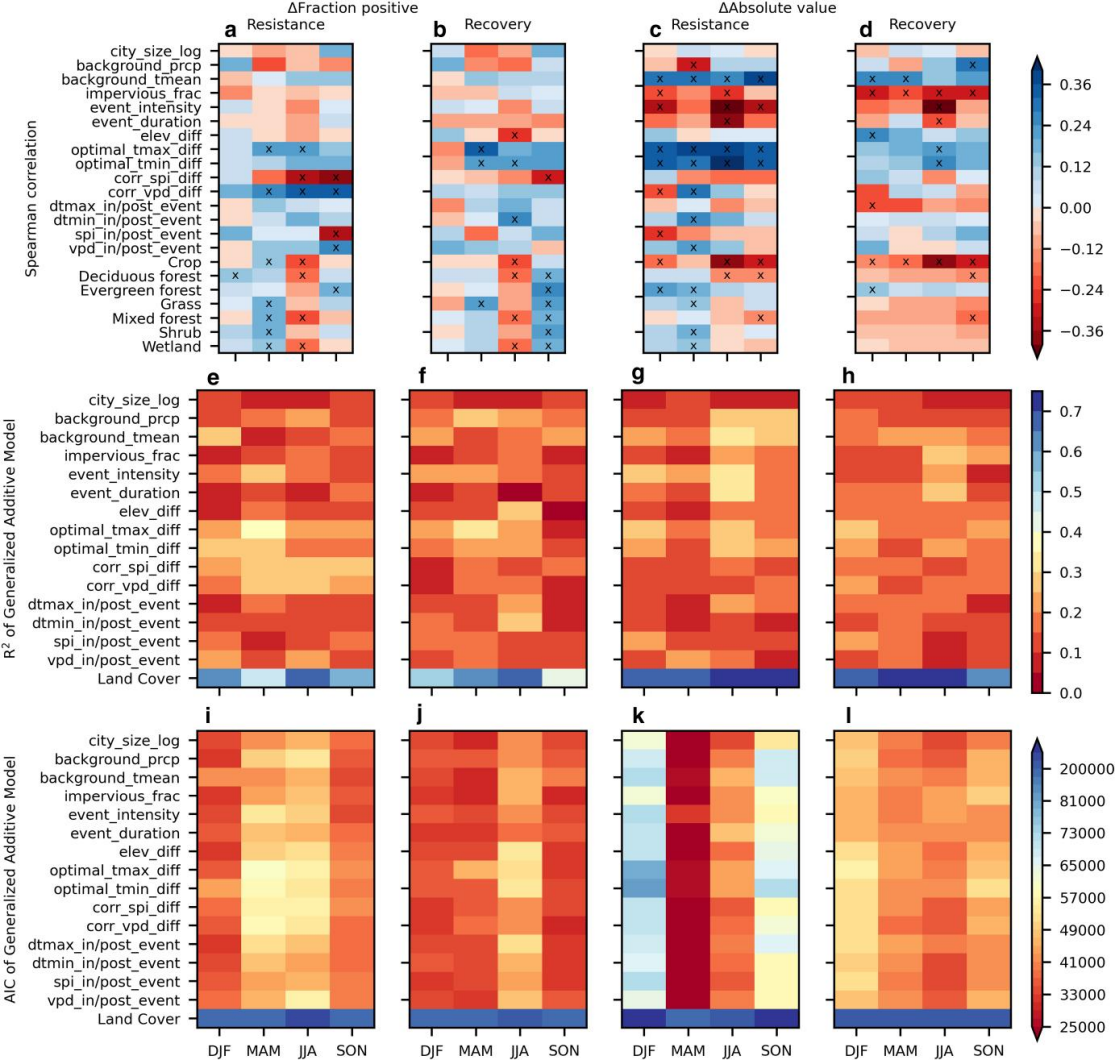
Spearman/partial Spearman correlations (a–d), *R*^2^ from GAMs (e–h), and AIC from GAMs (i–l) between various predictors and the urban–rural differences in the sign (Δ fraction positive) and magnitude (Δ absolute value) of resistances and recoveries. The partial Spearman correlations were for the land cover predictors (Deciduous Forest, Evergreen Forest, Grass, Mixed Forest, Shrub, Wetland). “X” on top of a Spearman/partial Spearman correlation means it was significantly different from zero at *P* ≤ 0.05. The GAMs used all the land cover predictors in one regression (labeled “Land Cover” in e–l), and the other predictors individually. Season abbreviations: DJF—December to February, MAM—March to May, JJA—June to August, SON—September to November. Exact details on the calculation of all quantities are in the supplementary material.

To better understand the drivers underlying the urban–rural differences found in Fig. 2 and Table S3, we used Spearman (for nonland cover predictors) and partial Spearman (for land-cover predictors) correlations, and the *R*^2^ and Akaike information criteria (AIC) of nonlinear generalized additive models (GAMs). Justification for the choice of those techniques and implementation details are in SI Section 1.2.4. Table S4 lists all the examined predictors and their definitions. Note the land cover predictors (Crop, Deciduous Forest, Evergreen Forest, Grass, Mixed Forest, Shrub, Wetland) were rural area fractions only, due to the lack of urban vegetation type information at the contiguous United States level (see SI Section 1.2.4 and discussion in the Limitations and outlook section). Therefore, the land cover predictors reflect the background biomes in which the cities are located, not urban–rural difference in vegetation types. Also note that although we excluded all the pixels that had ≥ 50% croplands cover from the analysis (SI Section 1.1.4), croplands still turned out to be more abundant than other land cover types in the rural areas of some cities, resulting in a Crop type in this section (Figs. 3 and S6).

For the urban–rural differences in sign, only the rural fraction of Deciduous Forest had significant partial Spearman correlation in winter (Fig. 3a). In spring, summer, and autumn, the urban–rural differences in the water stress status of the vegetation (corr_spi_diff, corr_vpd_diff) generally had significant correlations with the urban–rural differences in the sign of resistances, but not with recoveries (Fig. 3a and b). The urban–rural differences in the daytime and nighttime optimal temperature of vegetation growth (optimal_tmax_diff, optimal_tmin_diff, Fig. 3a and b) often had significant correlations for the signs of both resistances and recoveries (Fig. 3a and b). Those four predictors (corr_spi_diff, corr_vpd_diff, optimal_tmax_diff, optimal_tmin_diff) also had higher *R*^2^ (20–40%) and similar AICs to the other nonland cover predictors in the GAMs, further demonstrating their good explanatory power (Fig. 3e and f). The signs of the correlations were negative for corr_spi_diff, and positive for corr_vpd_diff (Fig. 3a and b), suggesting that where the urban vegetation were more water stressed than the rural vegetation, the urban vegetation were less enhanced/more impaired by hot months than rural vegetation. The signs of the correlations for optimal_tmax_diff and optimal_tmin_diff were positive in spring, summer, and autumn, meaning that where the urban optimal temperatures were higher, the urban vegetation responded better to hot months than rural vegetation. Based on the spatial patterns of those important predictors (Fig. S4a–j), one can further deduct that they all implied lower urban resilience in the eastern United States and higher in the western United States, consistent with the findings in Table S3. Sporadic strong correlations existed for urban–rural difference in elevation (elev_diff) (Fig. 3b) and moisture conditions during the hot months (spi_in/post_event, vpd_in/post_event) (Fig. 3a), but those had lower *R*^2^ values than the water stress status or optimal temperature predictors (Fig. 3e and f).

The land cover predictors generally had significant partial Spearman correlations in spring and summer for the urban–rural differences in the sign of resistances, and in winter for the sign of recoveries (Fig. 3a and b). Unexpectedly, the significant partial correlations always had the same signs for all land cover types in each season. Because the spatial distributions of rural land cover types were anticorrelated (Fig. S5b and c), if a land cover type was an important explainer of the urban–rural differences, its correlations should have opposite signs to its anticorrelated land cover types. Therefore, the identical signs suggest the correlation coefficients may be unreliable. Also, though the land cover-based GAMs generally had much higher *R*^2^ than the GAMs for nonland cover predictors, they also had much higher AIC (Fig. 3e, f, i, and j). Those results further suggest that the higher *R*^2^ were due to overfitting, which may have affected the partial correlation coefficients.

For the urban–rural differences in magnitude, the differences in the optimal temperatures of vegetation growth (optimal_tmax_diff, optimal_tmin_diff) and the climatological seasonal mean temperature over the whole city (background_tmean) showed significant positive correlations for resistances (Fig. 3c). The intensity of the hot months (event_intensity) showed significant negative correlations in three seasons for resistances (Fig. 3c). A few other predictors showed significant correlations in one or two seasons for resistances (Fig. 3c). The impervious fraction of the urban areas (impervious_frac) showed significantly negative correlations in all four seasons for recoveries, followed by the climatological seasonal mean temperature (background_tmean) in two seasons, and a few other predictors in one season (Fig. 3d). Among the predictors with significant Spearman correlations, background_tmean, event_intensity, and the duration of the hot months (event_duration) reached the highest *R*^2^ in summer (35–40%), followed by optimal_tmax_diff and optimal_tmin_diff in summer (30–35%) (Fig. 3g and h). Those results suggested that, compared to the urban–rural differences in sign, water stress status of the vegetation (corr_spi_diff, corr_vpd_diff) was less important, while temperature-related factors (optimal_tmax_diff, optimal_tmax_diff, background_tmean, event_intensity, event_duration) and impervious fraction (impervious_frac) were more important for urban–rural differences in magnitude. The signs of the correlations implied greater urban–rural similarity in magnitude toward the western and southern United States, where the urban–rural differences in optimal temperatures were higher, the climate warmer, and the hot months less intense (Fig. S4i–n). Urban impervious fraction also showed east–west contrast, but with the implication of greater urban–rural difference in magnitude toward the western United States (Fig. S5d).

Compared to urban–rural differences in sign (Fig. 3a and b), the partial Spearman correlations of the land cover predictors with urban–rural differences in magnitudes had fewer significances, but always exhibited both positive and negative correlations in the same season (Fig. 3c and d), and the *R*^2^ values of the GAMs were very high (around 0.7) (Fig. 3g and h). Therefore, although the AICs of the land-cover GAMs were still much higher than the single-predictor GAMs, land cover types seemed to have explanatory power for the urban–rural differences in magnitude. Higher rural Crop fractions implied significantly more negative urban–rural differences (i.e. less similarity between the urban and rural areas) in nearly all the seasons for both resistances and recoveries, and higher rural Deciduous Forest fractions had similar but smaller effects (Fig. 3c and d). Higher rural Evergreen Forest fractions tended to imply less negative urban–rural differences (i.e. more similarity between the urban and rural areas) (Fig. 3c and d).

To further understand the effects of land cover types on large-scale patterns in urban–rural differences, we performed urban–rural comparisons for subsets of cities that have the same dominant rural land cover types (Fig. S5b). The clearest urban–rural differences occurred in the Deciduous Forest subset, with the urban areas having significantly more positive responses in winter, significantly more negative recoveries in spring, and significantly smaller response magnitudes in all the seasons (Fig. S6e–h). For the Crop and Grass subsets, the urban–rural differences in magnitude were also consistently negative and especially large for the Crop subset, albeit with fewer statistical significance than the Deciduous Forest subset (Fig. S6a–d and m–p). For the Evergreen Forest subset, the urban–rural differences in sign were insignificant but had a clear seasonal cycle with positive differences in winter and autumn and negative differences in spring and summer, and the urban–rural differences in magnitude were the smallest among all the subsets (Fig. S6i–l). The greater number of statistical significances among the land cover subsets for urban–rural differences in magnitude than for sign (Fig. S6) support the idea that land cover was more important for magnitude differences than for sign differences (Fig. 3). Explanation of the predictors is shown in Table S4.

### Pixel-level controls of urban–rural differences in resilience metrics

To complement the above large-scale analysis using relatively simple regression techniques, we selected subregions of the contiguous United States to perform random forest classifications (for the signs of the responses) and regressions (for the magnitudes of the responses) to understand the controls of urban–rural differences at smaller spatial scales and the potential nonlinear and interactive effects of the predictors. We defined the subregions using “land cover groups,” i.e. cities with the same dominant rural land cover type, like the subsets in the Large-scale controls of urban–rural differences in resilience metrics section, but further restricted to the noncrop land cover types and cities in similar climate regions (Fig. S5c). Detailed descriptions and justifications are in SI Section 1.2.5.

**Fig. 4.**
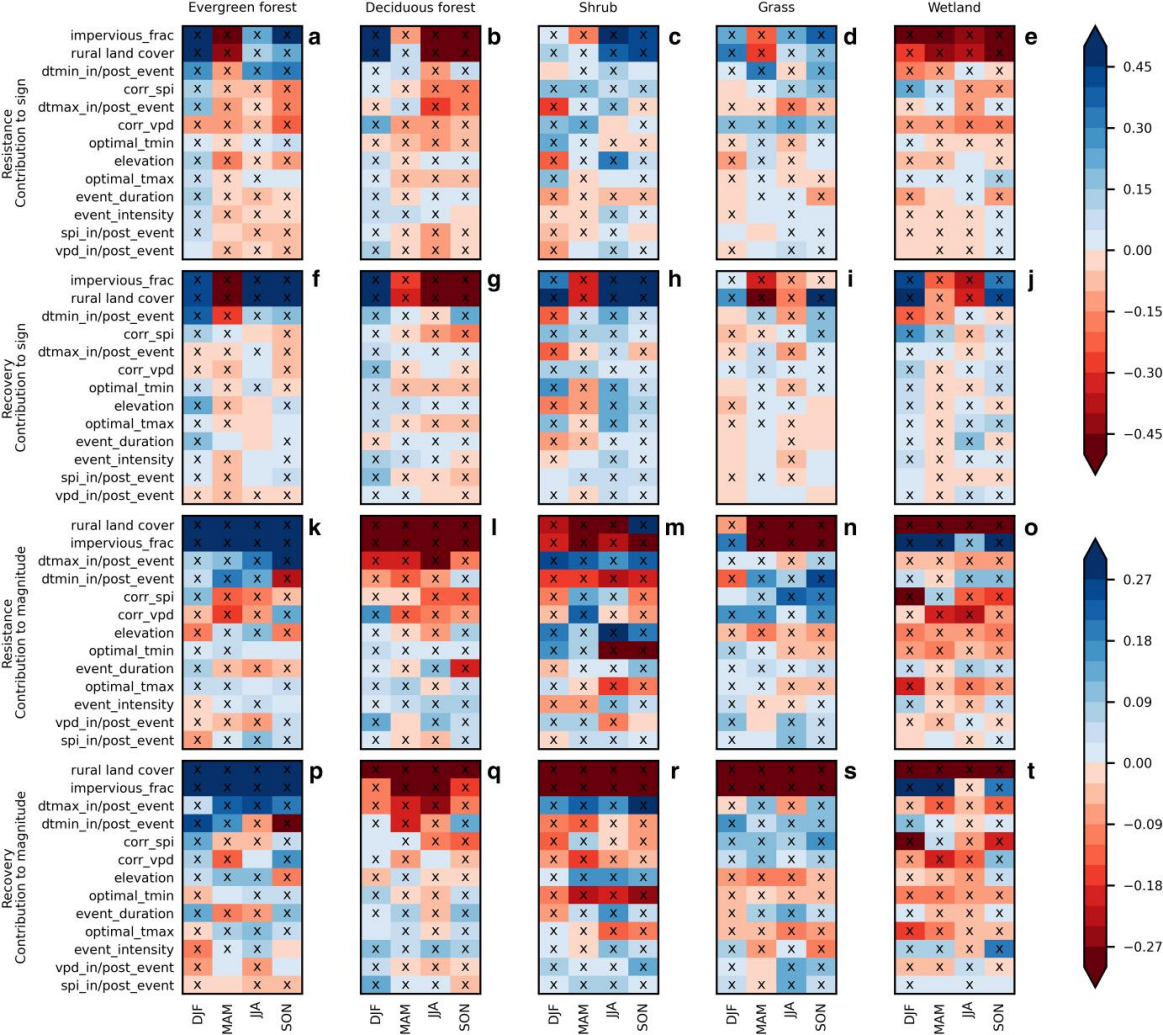
Contribution of various predictors to the sign (a–j) and magnitude (k–t) of the urban–rural differences in resistances (a–e, k–o) and recoveries (f–j, p–t) according to pixel-level regression by land cover groups. The predictors are arranged, separately for sign and for magnitude, in decreasing order of the average rank of absolute magnitude of contributions across all the seasons and land cover groups along the *y*-axis. The “rural land cover” refers to the dominant rural land cover of each land cover group and nondominant rural land cover types in each land cover group are omitted. Significant contributions (Spearman correlations) were denoted by “X”. Season abbreviations: DJF—December to February, MAM—March to May, JJA—June to August, SON—September to November. Details on the calculation of all quantities are in SI Section 1.2.5. Explanation of the predictors is shown in Table S5.

For urban–rural differences in both the sign and the magnitude of the responses, the most important (i.e. having the largest absolute contributions to urban–rural differences) predictors were the fraction of dominant rural land cover and the impervious fraction in the pixel (Fig. 4, note the predictors are arranged in decreasing order of importance). Differences across land cover groups were also notable. For urban–rural differences in sign, those two most important predictors had mainly positive contributions (i.e. higher urban responses compared to the rural baseline) in the Evergreen Forest and Shrub land cover groups, negative contributions (i.e. lower urban responses compared to the rural baseline) in the Deciduous Forest and Wetland groups, and mixed contributions in the Grass group (Fig. 4). With the abundance of Shrub in the western United States (Evergreen Forest was a small group), and the abundance of Deciduous Forest and Wetland in the eastern United States (Fig. S5), those contributions were broadly consistent with the east–west contrast in urban–rural differences in sign (Table S3). Those contributions were not consistent with the urban–rural differences across the subsets in Fig. S6, consistent with the previous interpretation that land cover was probably not a good explainer of urban–rural differences in sign (the Large-scale controls of urban–rural differences in resilience metrics section). For urban–rural differences in magnitude, the two most important predictors had mainly positive contributions (i.e. greater urban–rural similarity) in the Evergreen Forest group, mixed contributions in the Wetland group, and negative contributions (i.e. greater urban–rural disparity) in the other groups (Fig. 4). Those were consistent with the positive partial Spearman correlations in of Evergreen Forest in Fig. 3, and the greater urban–rural similarity in the Evergreen Forest and Wetland subsets than the other subsets in Fig. S6. This supports the previous interpretation that land cover had actual effects on urban–rural differences in magnitude (the Large-scale controls of urban–rural differences in resilience metrics section).

For urban–rural differences in sign (Fig. 4a–j), the third and fifth most important predictors were, respectively, nighttime and daytime urban heat island intensity (dtmin_in/post_event, dtmax_in/post-event). The dtmin_in/post_event predictor contributed to higher urban responses compared to rural in all the land cover groups except Wetland. These contributions were consistent with the facts that the urban–rural differences in dtmin_in/post_event were generally positive (Fig. S4y–ff) and that higher dtmin_in/post_event mainly led to more positive vegetation responses except in the Wetland land cover group (Fig. S9k–t). The water stress status for vegetation growth (corr_spi, corr_vpd), which were the most important in the city-level regressions (Fig. 3), ranked the fourth and sixth (Fig. 4a–j). Both corr_spi and corr_vpd mainly contributed to higher urban resistances compared to rural in the Shrub and Grass land cover groups, but lower urban resistances in the other groups (Fig. 4a–e). The contributions of corr_spi and corr_vpd to urban–rural differences in the sign of recovery were mostly weak (Fig. 4f–j). The contributions of corr_vpd were consistent with the facts that higher corr_vpd led to more positive resistances in all the land cover groups but mixed recoveries (Fig. S9k–t), and that the urban–rural differences in corr_vpd tended to be more positive toward the western United States, where Shrub and Grass are more dominant (Figs. S4 and S5). Similarly, the contributions of corr_spi were consistent with the facts that higher corr_spi mainly led to more negative resistance (Fig. S9k–t), and that the urban–rural differences in corr_spi tended to be more negative toward the western United States (Fig. S4). In the Shrub land cover group, elevation and the daytime and nighttime optimal temperature for vegetation growth (optimal_tmax, optimal_tmin) were important contributors in summer, likely due to the large urban–rural differences in elevation and optimal temperatures in the southwestern United States (Figs. S4 and S5).

For the urban–rural differences in magnitude (Fig. 4k–t), the third and fourth most important predictors were the daytime and nighttime urban heat island effects (dtmax_in/post_event, dtmin_in/post_event). Both mainly contributed to greater urban–rural similarity in the Evergreen Forest and Grass land cover groups, and mainly greater disparity in the Deciduous Forest group. The two had opposite signs of contribution in the Shrub and Wetland groups. These can be compared with the facts that the pixels with higher dtmax_in/post_event and dtmin_in/post_event had larger magnitudes of responses in the Evergreen Forest and Grass groups (Fig. S10k, n, p, and s) and smaller magnitudes in the Deciduous Forest group (Fig. S10l and q). In the Shrub group, higher dtmax_in/post_event led to larger magnitudes of response, but higher dtmin_in/post_event led to smaller magnitudes of response (Fig. S10m and r). In the Wetland group, the opposite signs of contribution between dtmax_in/post_event and dtmin_in/post_event were consistent with the fact that the daytime urban heat island was negative in some cities in Florida, where the Wetland land cover is dominant, but the nighttime urban heat island was not (Figs. S4q–ff and S5). The fifth and sixth most important predictors were the water stress status predictors (corr_spi, corr_vpd). Both mainly contributed to greater urban–rural similarity in the Grass group, and mainly greater disparity in the other land cover groups (Fig. 4k–t). This pattern was not entirely consistent with the spatial distributions of the urban–rural differences in corr_spi and corr_vpd (Fig. S4), and the partial dependence relationships between corr_spi/corr_vpd and the magnitude of the resilience metrics (Fig. S10), suggesting the existence of interactive effects with other predictors. Likely due to the same reasons as for the urban–rural differences in sign, elevation and the nighttime optimal temperature for vegetation growth (optimal_tmin) were important predictors in the Shrub land cover group (Fig. 4m and r).

## Discussion

### Patterns of the urban and rural resilience metrics

We found that hot months broadly induced positive concurrent changes in vegetation greenness but mixed signs of delayed responses, with especially more negative delayed responses in spring and summer (Fig. 1). EVI is positively correlated with vegetation cover and gross primary productivity (35–37). Therefore, the findings suggest that hot months probably enhanced vegetation growth in both the urban and rural areas of the contiguous United States during the colder seasons, but mainly hindered the two with a time lag during the warmer seasons.

Consistent with previous studies, the responses during the hot months were more positive in the colder and more humid parts of the United States than in the warmer and drier parts, and more positive in the spring than in the warm summer season or in the autumn senescence season (Fig. 1) (13, 14, 20). Such patterns were consistent with the expectations that in the seasons and regions where vegetation growth is temperature limited, warm extremes can promote growth by alleviating this limitation, whereas in the seasons and regions where vegetation growth is water-limited, warm extremes can impair growth by exceeding the optimal growth temperature or by increasing the transpiration demand.

The existence of negative delayed responses supports the existence of oscillatory behavior after disturbance observed in a past study (38). To verify the negative recoveries were not due to random noise, we examined the relationship between resistances and recoveries using the median values of the urban (or rural) areas over all the hot months, cities, and meteorological datasets. Resistances were strongly positively correlated with recoveries in winter, spring, and autumn, but not in summer, where many events caused strong positive resistances but strong negative recoveries (Fig. S11a–h). Therefore, at least the summer negative recoveries were unlikely due to random noise. Furthermore, the summer negative recoveries were especially common for long and intense events (duration ≥ 4 months, intensity ≥2 °C) (Fig. S11i–p). Those results supported the previous finding that strong warm periods can cause delayed negative vegetation responses even months after, but whereas the previous finding applied to spring, our finding applied to summer (19, 20). Longer and more intense warm extremes have greater possibility of inducing drought through land–atmosphere feedbacks and of depleting the stored carbohydrates and soil moisture through enhanced leaf area expansion, which may explain the 4-month, 2 °C thresholds found in this study (19, 39).

### Seasonal variations in the urban–rural differences in resilience metrics

We found the urban–rural differences in resistance to be similar to that of recovery. The similarity might be because the signs and magnitudes of recoveries were dependent on the signs and magnitudes of the resistances (Fig. S11). This include situations when negative recoveries followed positive resistances, because, for example, with less concurrent enhancement of vegetation growth in the urban areas, the soil moisture depletion would be less severe, resulting in less delayed impairment on vegetation growth.

In winter, the generally more positive urban than rural responses (Fig. 2; Table S3) may be because phenological changes require reaching a certain level of cumulative temperatures (40), and the urban heat island made it easier for vegetation to reach this threshold during hot months. Additionally, the especially more positive urban resistances in the Deciduous Forest biome (Fig. S6e and f) might be because the urban vegetation retained more leaves than the rural vegetation in winter, due to human curation of species or management advantages.

In spring, summer, and autumn, the generally more negative urban than rural responses in the eastern United States (Table S3) are consistent with the expectation from urban heat island effect. Higher urban than rural air temperatures would make the urban vegetation experience more heat stress than the rural vegetation during the same city-scale hot months. In the western United States, however, urban responses tended to be more positive (Table S3), despite the stronger urban heat island in the western than the eastern United States (Fig. S4s–hh). Regression results suggest this was caused by lower water stress status of the urban vegetation and higher optimal temperature of growth (Figs. 3 and S4a–j). The lower water stress and high optimal growth temperature of urban vegetation in the western United States were likely caused by irrigation. In the western United States, water imported to a basin and used in outdoor landscaping can consume up to 5 times the naturally available water in some arid urban areas (28, 41, 42). The greater water availability for the urban vegetation may have enabled stronger cooling through evapotranspiration, and hence continued growth under higher temperatures than the rural vegetation (11, 43, 44). Other reasons behind the mostly higher optimal temperature of growth of urban vegetation (Fig. S4i–j) could be thermal acclimation, evolutionary pressure, and human selected and invasive species (23, 45, 46). But thermal acclimation was perhaps not a main cause in this study, because it would imply higher urban–rural differences in optimal temperatures where the urban heat island intensities were higher. Instead, patterns in the eastern United States show lower urban–rural differences in optimal temperatures where the urban heat island intensities were higher (Fig. S4i–j and s–hh). In all the seasons, the generally smaller magnitudes of the urban resilience (Fig. 2) may be because human management activities (e.g. irrigation, fertilization) damped the effects of natural weather variations.

### Moisture and temperature controls on the urban–rural differences in resilience metrics

As noted above in explaining the east–west contrast, the water stress status of vegetation was an important factor in explaining the urban–rural differences in the sign of the resilience metrics (corr_vpd_diff, corr_spi_diff in Fig. 3). Within individual land cover groups, water stress status was less important to the urban–rural differences than land cover and temperature factors, but still relatively important (corr_vpd, corr_spi in Fig. 4). The smaller scale contributions of water stress status to urban–rural differences were either positive or negative (corr_spi, corr_vpd in Fig. 4a–j), consistent with the fact that the urban–rural differences in water stress status had spatially varying signs (Figs. S4a–h and S5b). As such, those findings did not support previous findings that urban vegetation may be more water stressed because of limited rooting space and soil compaction (47). Established urban trees may have access to water in the sewage system through their deeper roots (48), while smaller trees and trees planted in restricted spaces may be more water-limited.

In both the city-level and the pixel-level regressions, temperature-related predictors were important to both the sign and magnitude of the responses, but the specific predictors differed. In the city-level regressions, the urban–rural differences in optimal temperature (optimal_tmax_diff, optimal_tmin_diff), and, for urban–rural differences in the magnitude of the resilience metrics only, the seasonal climatology of the city-average temperature (background_tmean) and the intensity of the hot months (event_intensity) were of high importance (Fig. 3). The climatological temperature had a north–south spatial pattern that differed from other important predictors of urban–rural differences (water stress status, urban heat island intensity, and land cover types [Figs. S4 and S5]), suggesting its effect was through a distinct mechanism, not confounding effects. The hot months did become less intense toward the southern and western United States (Fig. S4o–r), implying potential confounding effects with the climatological temperature. In the pixel-level regressions, the most important temperature-related predictor was local urban heat island intensity (dtmax_in/post_event, dtmin_in/post_event) (Fig. 4). This is in contrast to the greater importance of optimal growth temperatures in the city-level regressions. Considering that within the same climate region, urban irrigation levels are likely to be similar, the pixel-to-pixel variations in optimal growth temperatures within each land cover group of Fig. 4 may therefore be less than the pixel-to-pixel variations in local urban heat island intensity. This would explain the greater importance of the urban heat island effect in Fig. 4.

Interestingly, stronger local urban heat island intensity, especially at night, often led to more positive resilience metrics in the Evergreen Forest, Deciduous Forest, Shrub, and Grass land cover groups, despite the implied greater likelihood of exceeding optimal temperature (Figs. 4a–j and S9k–t). This may be in part because warmer nighttime temperatures can reduce frost damage to leaves during the phenological transition seasons (49, 50). Another factor may be acclimatization in photosynthesis and respiration in the warmer parts of the city (51, 52), which has the possibility of being transient and hence not reflected in the optimal temperatures calculated from multiyear data at annual level (SI Section 1.2.4) (53). Also, the warmer parts of a city may be more built-up, with more nighttime lighting that alters vegetation phenology (28, 54). The contributions of local urban heat island intensity to the magnitude of the resilience metrics depended strongly on the land cover groups (Fig. 4k–t). This may be because the shapes of the temperature response curves differed between vegetation types (55, 56).

### Land cover and impervious area controls on the urban–rural differences in resilience metrics

In the city-level regressions, land cover type explained well the urban–rural differences in both the sign and the magnitude of the responses, but at the cost of overfitting, especially for the sign, compared to single-predictor regressions (Fig. 3). In the pixel-level regressions, the fraction of the dominant rural land cover and impervious area were of primary importance in explaining the urban–rural differences in both the sign and the magnitude of the responses, with the direction of the effects showing interactions with the dominant rural land cover types (Fig. 4).

The effects of impervious fraction may be related to a wide range of factors (e.g. prevention of infiltration, radiative effect of impervious surfaces, elevated CO_2_, air pollution, disturbance levels, physiological changes in vegetation, irrigation, fertilization) in both the eastern and western United States (28, 30, 57, 57–62). Except when the rural area is dominated by Evergreen Forest, higher impervious fraction mainly decreased the sign and magnitude of the urban responses (Fig. 4). This is somewhat contrary to previous findings that urban development enhanced maximum vegetation greenness (63, 64) and suggests that urban vegetation may be unable to take advantage of the beneficial factors in the urban environment during hot months.

Past research indicated that land cover type can explain how vegetation respond to hot months across large regions, but did not use a metric-like AIC to compare between the land cover and nonland cover factors, controlling for overfitting (13–16). As such, the effect sizes might be overestimated because of the covariation between land cover and prevailing climate over large regions. There is no doubt that a land cover effect exists, which is necessitated by plant- and site-scale mechanisms (65–67). Our city- and pixel-level regression results also supported the existence of such an effect for the magnitude of vegetation response (Figs. 3, 4, and S6). Future studies are needed to more accurately separate the effects of land cover from that of the prevailing climate and other spatially covarying factors (e.g. some of the factors in Figs. S4 and S5) at large scale.

The magnitudes of the urban and rural responses were most similar in the Evergreen Forest land cover group, least similar in the Deciduous Forest land cover group, and had intermediate differences for the other land cover groups (Figs. 3, 4, and S6). The differences between Evergreen Forest and the other land cover groups may be because in the southeastern United States, the evergreen forest species were mainly gymnosperms (loblolly and short leaf pines) (68). Gymnosperms have more conservative water use strategy that made them less sensitive to drought than angiosperms (7). Given the important role of moisture (Moisture and temperature controls on the urban–rural differences in resilience metrics section), the same mechanisms might have caused the gymnosperms to be less sensitive to hot months, and thus exhibit similar or even lower magnitudes of resilience than the urban vegetation (Figs. 4k, p and S6k, l). Another study also found that deciduous broadleaf forests responded more positively to higher air temperatures in summer than evergreen needleleaf forests (69). The open canopy of grasslands and shrublands make the soil prone to drought during hot months (14), which, in combination with urban irrigation in the western United States, may explain why the resilience became more positive toward more urbanized areas for the Shrub and Grass land cover groups (Figs. 4c, d, h, l and S6m, n, u, v).

### Limitations and outlook

Some limitations exist in the methodology and data of this study. There is especially a need for more comprehensive and accurate urban datasets for understanding urban vegetation resilience in the future. We detail the limitations as follows.

We delineated the seasons using the conventional 3-month windows, but during the transition seasons of spring and autumn, temperature shifts as much as 7–14 °C across the contiguous United States except along the western and Gulf coastlines. Vegetation undergo phenological transitions during those seasons, with the exact timing varying across the United States due to differences in biome and climate, and phenological stages can affect plant resilience to stressors (70). Therefore, the conventional seasonal delineation may have obscured the exact timing of transition between mainly positive and mainly negative recoveries, resulting in the apparent spatial shift of the cluster of negative recoveries from southeastern United States to midwestern United States from spring to summer (Fig. 1j, k, n, and o). Improved seasonal delineations, such as based on vegetation phenological stages or catered to local temperature seasonality, may generate more homogenous spatial patterns.

In all the interpretations and regressions, we treated vegetation growth as a consequence of hot months and did not account for feedback effects (71). The feedback effect of urban vegetation on temperature is cooling (72, 73). Therefore, this limitation cannot invalidate our findings that the vegetation responds mainly positively to hot months (Fig. 1) and higher local urban heat island intensity often positively affect the resilience metrics (Figs. 4 and S9), but may have affected the detailed values and statistical significance of the regression results. Future work may obtain more accurate results by using statistical frameworks that can account for bidirectional effects, such as structural equation modeling (74). Also, our regressions did not explicitly consider cumulative effects across consecutive hot months, although partially implicitly accounted for the effect through the concatenation of hot months separated by <3 months (SI Section 1.2.1). Between hot months, moderately high temperatures below the 90th percentile threshold (SI Section 1.2.1) may induce greater heat tolerance in the plants through the priming effect (12, 67) or lower heat tolerance through continued soil moisture drawdown (75). Therefore, future studies may want to explore the cumulative effects further.

We interpreted the correlation with SPI and VPD as indicating the water stress status of vegetation, which is inexact (76) and may be the reason why our identified patterns of urban–rural differences in water stress status were very heterogeneous (Figs. S4a–h). More direct indicators like soil moisture and vegetation optical depth are currently only widely available at tens of kilometers resolution and the retrieved values are inaccurate in urban areas (77–79). Soil moisture and vegetation monitoring with remote sensing exhibit great uncertainties in urban areas because of heterogeneous land cover within the pixels (e.g. building, shade, road, and vegetation), more diverse tree species, heterogeneous soil properties, and stronger radio-frequency interference (80–82). Therefore, future studies should explore an integration of multisensor remote sensing (e.g. optical, microwave, hyperspectral, and active light detection and ranging [LiDAR]) with process-based models and local municipal data repositories to create accurate datasets on urban vegetation water status (81, 83, 84).

We chose EVI, instead of more direct measures of vegetation growth like leaf area index or gross primary productivity, to perform the analysis because global datasets for those latter variables either did not have values or were not well validated over urban areas (85–88). Also, none of those vegetation indices can reveal changes in vegetation types, e.g. replacement of woody vegetation by crops or grass (89–91). If such a replacement occurs, urban ecosystem services may decrease despite an increase in EVI during hot months because trees often provide greater benefits than grasses (65, 92, 93). Urban vegetation resilience are additionally complicated by factors such as diverse tree species that have opposite responses to hot months (94), and private lawns and golf courses being better irrigated than street trees or urban parks. To understand those unknowns require detailed land cover imagery at 1 m or higher resolution, which have started to become available over smaller spatial areas during the past 10 years (95–97).

In order to enable considering uncertainty in air temperatures by using three meteorological datasets, we used only one EVI dataset (98, 99) and only one dataset for SPI and VPD (Daymet), which may result in underestimation of the uncertainty in input data. The baseline period of the hot months was restricted to 2001–2019, which was slightly shorter than the more desirable 30-year climatological period. We also lacked the data (e.g. CO_2_ concentration, air pollutant levels, soil hydrology) to determine the mechanisms behind the effect of impervious fractions on vegetation growth. Those limitations can be resolved as future studies develop more and better urban meteorological datasets beyond temperature, gap-filled EVI datasets, and urban air quality and soil data with comprehensive spatial coverage through an integration of ground sampling, remote sensing, and modeling (100–102).

## Conclusions

We calculated and compared the response of urban and rural vegetation greenness (EVI during 2001–2019) to hot months at the monthly time scale for 85 major cities in the contiguous United States, using one metric for concurrent response (resistance) and one metric for delayed response (recovery). We found hot months induced generally positive concurrent responses and both positive and negative delayed responses for both urban and rural vegetation. Negative delayed responses were especially prone to occur in summer after hot months that were longer than 4 months and more intense than 2 °C above the climate normal. In winter, vegetation in the warmer urban areas responded more positively to hot months than rural vegetation. In the other seasons, urban vegetation responded more positively in the western United States, but more negative in the urban areas in the eastern United States, than the rural areas. The east–west pattern can be explained by the relatively high optimal temperatures and low water stress of urban vegetation in the west, which may be related to urban irrigation in the western United States. Urban vegetation in general appeared to be less perturbed by hot months than rural vegetation, with the magnitudes of the responses being smaller in all the seasons and both the eastern and western United States Land cover type also mattered to the magnitude of the response. The magnitude of the urban responses was most similar to rural evergreen forests and least similar to rural deciduous forests. Pixel-level regressions within groups of cities that had similar climate and rural land cover types showed that the pixel-to-pixel variations in impervious area, vegetation fraction, local urban heat island intensity, and water stress status were the key small-scale factors that gave rise to the city-scale urban–rural differences.

These findings contribute to scientific knowledge by showing that temperature, moisture, vegetation type, and impervious area factors were important to large- and small-scale variations in vegetation resilience to hot months, adding to a line of literature on urban–rural differences in vegetation behaviors (29–32). The findings also have implications for urban forestry and ecosystem management (47, 103). For example, the delayed negative responses suggest that monitoring of vegetation health is important after warm extremes, and that cost-effective prevention of negative impacts from hot months may be achieved by focusing on the longest and most intense hot months. The importance of moisture conditions suggests that promoting urban vegetation growth requires not only managing the urban heat island but also the urban dry/wet island (104). In the future, dataset development efforts are needed to better understand the vegetation physiological and structural changes underlying the observed greenness changes during and after the hot months. In this regard, urban foresters and ecosystem managers are uniquely positioned in their access to detailed municipal level data (e.g. tree species, LiDAR maps, soil surveys, and air quality stations). Such data will help them understand ground changes during hot months that cannot be resolved by remote sensing information over large regions, making better decisions such as on selection of tree species and issuing land development permits, and quantification of ecosystem services in relation to remotely sensed vegetation changes.

## Data and methods

We used Eqs. 1 and 2 to calculate resistance and recovery from the periods of extreme warm monthly mean temperatures (“hot months”) and deseasonalized monthly EVI data, at 1 km pixel level in all the cities. The subscripts “in,” “pre,” and “post” mean, respectively, during, before, and after the hot months. The analysis period spans 2001–2019. We used three different sources of air temperature to identify the hot months, and used gap-filled MODIS EVI data from the North American Carbon Program (98, 99, 105–107). We further analyzed the relationships between the urban–rural differences in resistance/recovery and the water, thermal, land cover, and elevation environment using various auxiliary variables. Details on the data sources and data preparation are in SI Section 1.1. Detailed information on the statistical tests to determine the significance of urban–rural differences, and the regression and feature importance methods to assess relationships between urban–rural differences and the auxiliary variables, are in SI Section 1.2:


(1) $$\mathrm{Resistance}=\frac{2\times (\mathrm{EV}I_{\mathrm{in}}-\mathrm{EV}I_{\mathrm{pre}})}{\left(\mathrm{EV}I_{\mathrm{pre}}+|\mathrm{EV}I_{\mathrm{in}}-\mathrm{EV}I_{\mathrm{pre}}|\right)}$$

(2) $$\mathrm{Recovery}=\frac{2\times (\mathrm{EV}I_{\mathrm{post}}-\mathrm{EV}I_{\mathrm{pre}})}{\left(\mathrm{EV}I_{\mathrm{pre}}+|\mathrm{EV}I_{\mathrm{post}}-\mathrm{EV}I_{\mathrm{pre}}|\right)}$$

## Supplementary Material

pgae147_Supplementary_Data

## Data Availability

The MOD09Q1G EVI data were downloaded from https://ladsweb.modaps.eosdis.nasa.gov/archive/allData/6/MOD09Q1G_EVI/. The Daymet v4 data were downloaded from https://daac.ornl.gov/DAYMET/guides/Daymet_Daily_V4.html. The TOPOWx data were downloaded using the climateR package at https://github.com/mikejohnson51/climateR. The Zhang22 data were downloaded from https://doi.org/10.25380/iastate.c.6005185. The NLCD land cover types and impervious fraction data were extracted using google earth engine from https://developers.google.com/earth-engine/datasets/catalog/USGS_NLCD_RELEASES_2019_REL_NLCD. The elevation data were extracted using google earth engine from https://developers.google.com/earth-engine/datasets/catalog/USGS_NED. The Supplementary material is available at veg_resilience_urban_rural_constrat_SI_04022023.docx. The code required to reproduce this work is available at https://github.com/ypwong22/urban_resilience.git.
