# Supplementary material for: Thermal, water, and land cover factors led to contrasting urban and rural vegetation resilience to extreme hot months
Source: PNAS Nexus. 2024 Apr 15;3(4):pgae147. doi: 10.1093/pnasnexus/pgae147 (PMC11026108; doi:10.1093/pnasnexus/pgae147)
Supplement: pgae147_Supplementary_Data [file pgae147_supplementary_data.docx]

Thermal, Water, and Land Cover Factors Led to Contrasting Urban and Rural Vegetation Resilience to Extreme Hot Months

Yaoping Wang^1,*^, Jiafu Mao^1,*, †^, Christa M. Brelsford^2,3^, Daniel M. Ricciuto^1^, Fengming Yuan^1^, Xiaoying Shi^1^, Deeksha Rastogi^4^, Melanie M. Mayes^1^, Shih-Chieh Kao^1^, Jeffrey M. Warren^1^, Natalie A. Griffiths^1^, Xinghua Cheng^5^, David J. Weston^6^, Yuyu Zhou^7^, Lianhong Gu^1^, and Peter E. Thornton^1^

*^1^ Environmental Sciences Division and Climate Change Science Institute, Oak Ridge National Laboratory, Oak Ridge, TN, USA, 37830*

*^2^ Geospatial Science and Human Security Division, Oak Ridge National Laboratory, Oak Ridge, TN, USA, 37830*

*^3^ Analytics, Intelligence and Technology Division, Los Alamos National Laboratory, Los Alamos, NM, USA, 87545*

*^4^ Computational Science and Engineering Division, Oak Ridge National Laboratory, Oak Ridge, TN, USA, 37830*

*^5^ Department of Natural Resources and the Environment, University of Connecticut, Storrs, CT, USA, 06269*

*^6^ Biosciences Division, Oak Ridge National Laboratory, Oak Ridge, TN, USA, 37830*

*^7^ Department of Geography, the University of Hong Kong, Hong Kong, China, 999077*

^†^ To whom correspondence should be addressed. Email: [maoj@ornl.gov](mailto:maoj@ornl.gov)

^**^These authors contributed equally to this work.

# Supplementary Text

## Data

### Meteorological datasets

To account for observational uncertainty in the timing and intensities of extremely warm periods, we utilized three meteorological datasets in this study: Daymet v4 (Daymet), TOPOWx, and a recently produced dataset by Zhang et al. (2022b) (Zhang22). Table S1 lists basic characteristics of these products. All three datasets used ground weather stations and elevation-based factors in the interpolation (1–3). The TOPOWx and Zhang22 datasets additionally used remotely sensed land surface temperature in the interpolation (1, 3). All three datasets adopted different interpolation algorithms (1–3). We resampled all three meteorological datasets to the 1 km grid of Daymet using nearest neighbor and averaged the daily maximum and minimum temperature values to monthly. Except when calculating the optimal temperature of vegetation growth (Sect. 1.2.4), we performed all the statistical tests, correlations, and regressions on each meteorological dataset separately, and showed only the median numbers (including the p-values) over the three datasets in the results to reflect consensus.

### Enhanced vegetation index

We focused on the Enhanced Vegetation Index (EVI) in this study because the index is not sensitive to soil background interference and has been extensively used in past studies on urban vegetation (4–6). With the short analysis period (post-2000s), there were only ~300 “hot months” events that met the threshold-based definition (Sect. 1.2.1) in each season and meteorological dataset. To make best use of this small number of events, we used a gap-filled EVI product, MOD09Q1G, at 8-day, 250 m resolution from the MODIS for North American Carbon Program (7, 8), so that resilience metrics could be calculated for each event (Table S1). We resampled the EVI data to the 1 km grid of Daymet using the nearest neighbor method and to monthly time step using the maximum composite method.

### Land cover, impervious fraction, and elevation

For the analysis of factors controlling urban-rural differences in the resilience metrics, we used the land cover and impervious fraction data from the National Land Cover Database 2019 (NLCD) at 30 m resolution and the elevation data from the National Elevation Dataset (NED) at 1/3 arc second (~10 m) resolution (9, 10). We resampled both datasets to the 1 km grid of Daymet by averaging all the NLCD or NED pixels that fell within each 1 km pixel. Originally, each NLCD pixel belongs to only one land cover class. Our resampled 1 km pixels contain percentage values that indicate how many NLCD pixels in this 1 km pixel belong to each land cover class. We also simplified the NLCD land cover classes by aggregation (Table S2). The NLCD dataset contains land cover change information at a frequency of every 2–3 years between 2001 and 2019 (Table S1). We therefore used linear interpolation between every 2–3 years to find the annual values between 2001 and 2019.

### Urban masks

We obtained urban areas from a Defense Meteorological Satellite Program/Operational Linescan System nighttime light data and focused on the 85 cities that are greater than 500 km^2^ because of their stronger urban heat island effects, following a previous study (11, 12) (Fig. S2). For each urban area, we obtained the corresponding rural area as a buffer zone around the urban area’s boundary that is 3 times the size of the urban area, based on similar previous approaches (12). The choice of this large rural buffer was to ensure it contains vegetation that is distant from the urban heat island. The selected cities cover all the major climate zones in the U.S., including tropical/subtropical, temperate, continental, mediterranean, and semi-arid/arid. The covered biomes include shrublands in the southwest, grasslands in the west and Great Plains, croplands in the Midwest, deciduous forest in the east, wetlands in the southeast, and evergreen and mixed forests throughout (Fig. S5b). The urban areas generally had much more built-up areas with impervious surfaces than the rural areas, with the greatest contrast occurring in the western U.S., and lowest contrast in the Midwest (see the “Developed” fractions in Fig. S5ab).

Since crop sowing and harvesting may create artificial resilience values, we excluded all the pixels with $\geq$50% crop land cover. Since resilience is meaningless for non-vegetated areas, we also excluded all the pixels with $\geq$40% water land cover or $\geq$80% impervious fraction. The crop, water, and impervious fraction percentages used here were the average percentages over 2001–2019 in the NLCD dataset. We also excluded, for each season of analysis, all the pixels where the seasonal average EVI were less than or equal to 0.05, using the seasonal average EVI over 2001–2019.

## Methods

### Extraction of the hot months

We defined hot months at the city level and the monthly time scale based on 90^th^ percentile thresholds for each month of the year. Specifically, for each city and each of the three meteorological datasets, we calculated the city-average monthly average daily maximum temperature time series, which included both urban and rural areas. Then, for each month of the year, we calculated the 90^th^ percentile of this series during the period when data existed in both the meteorological dataset and the EVI dataset (Daymet v4: 2001–2019; TOPOWx: 2001–2016; Zhang 22: 2003–2019). If a monthly temperature exceeded the 90^th^ percentile for that month of the year, we considered it to be part of a “hot months” event. Initially, we separated the events by at least one month of non-extreme temperature. However, given that vegetation recovery from an extreme event can take several months (13, 14), and because the EVI data contains uncertainty, one-month intervals may be too short to reveal the true vegetation recovery after the event. Therefore, in the second pass, we merged all the hot months that were separated by one or two months of non-extreme temperature. As a result, all final “hot months” events were separated by at least three months of non-extreme temperature. We tagged the season of an event based on the month in which it ended, regardless of when it started. Therefore, the last month of a winter event was in December, January, or February; the last month of a spring event was in March, April, or May; the last month of a summer event was in June, July, or August; and the last month of an autumn event was in September, October, or November.

### Calculation of the resilience metrics

For each “hot months” event in any of the three meteorological datasets, we calculated the resilience metrics for each 1 km pixel in each city. We used two resilience metrics, resistance (Eq. 1) and recovery (Eq. 2). Resistance measures the maximum departure of the EVI from the pre-event level during the event, while recovery measures the average departure of the EVI from the pre-event level during the three months immediately after the event. These indices were modified from those previously used in engineering resilience literature (15, 16) with the following considerations. The denominators are the sum of the pre-event EVI level ($\mathrm{EVI}_{\mathrm{pre}}$) and the magnitude of the disturbances ($\left| \mathrm{EVI}_{\mathrm{in}}-\mathrm{EVI}_{\mathrm{pre}} \right|$ or $\left| \mathrm{EVI}_{\mathrm{post}}-\mathrm{EVI}_{\mathrm{pre}} \right|$) to prevent infinitely large metrics when disturbances were small. More specifically, $\mathrm{EVI}_{\mathrm{pre}}$ is the average EVI value during a pre-event period that has the same length as the event, i.e., based on the mirroring approach (17). $\mathrm{EVI}_{\mathrm{in}}$ is the EVI value during the event that differs the most from the pre-event EVI level. We identified $\mathrm{EVI}_{\mathrm{in}}$ by calculating the absolute differences between all the EVIs during the event and the pre-event EVI level, finding the month in which the absolute differences reached their maximum, and using the EVI of that month as $\mathrm{EV}I_{\mathrm{in}}$. $\mathrm{EVI}_{\mathrm{post}}$ is the average EVI during the three months immediately following the event. As mentioned in Sect. 1.2.1, we chose the three months period as a trade-off between retaining a sufficient number of events after merging, and having a long enough post-event period for the vegetation recovery signal to emerge. We avoided normalizing the recovery using the during-event EVI level because past studies showed it could lead to mathematical artificiality (16). The numerators are actual, not absolute, values to reveal whether the hot months enhanced or impaired vegetation growth. The scaling factor 2 on the numerators ensures the metrics reach ±1 when the size of the disturbance is equal to the pre-event vegetation level. Because the resilience metrics were normalized, they were comparable between events in different years and pixels. To prevent the seasonal growth and senescence of vegetation from confounding the resilience metrics, we removed the seasonality from the monthly EVI series in each pixel before calculating $\mathrm{EVI}_{\mathrm{pre}}$, $\mathrm{EV}I_{\mathrm{in}}$, and $\mathrm{EVI}_{\mathrm{post}}$. The removal involved subtracting the monthly climatology and then adding back the annual climatology of the EVI during 2001–2019. We did not de-trend the EVI because we deemed the influence of any long-term trend to be negligible over the time span of individual events in this study (always ≤ 12 months). Fig. S1 gives an illustration of the calculation of the resistance and recovery metrics.

|  | $\mathrm{Resistance}=\frac{2\times\left( \mathrm{EVI}_{\mathrm{in}}-\mathrm{EVI}_{\mathrm{pre}} \right)}{\left( \mathrm{EVI}_{\mathrm{pre}}+\left\vert\mathrm{EVI}_{\mathrm{in}}-\mathrm{EVI}_{\mathrm{pre}} \right\vert\right)}$ | (1) |
| --- | --- | --- |
|  | $\mathrm{Recovery}=\frac{2\times\left( \mathrm{EVI}_{\mathrm{post}}-\mathrm{EVI}_{\mathrm{pre}} \right)}{\left( \mathrm{EVI}_{\mathrm{pre}}+\left\vert\mathrm{EVI}_{\mathrm{post}}-\mathrm{EVI}_{\mathrm{pre}} \right\vert\right)}$ | (2) |

### Statistical test of urban-rural differences in resilience metrics

We indexed the raw resilience metrics using the following four quantities: meteorological dataset, city, event (start and end time), and pixel in the city. The hot months were also labeled by the seasons that they ended in (see Sect. 1.2.1).

For each meteorological dataset, season, and city, we pooled the pixel-level raw resistances or recoveries into an urban set and a rural set. We used the Wilcoxon signed rank test (two-sided, paired test) to determine, for the urban set and the rural set separately, whether each was significantly different from zero (18). To determine whether the urban set and rural set differed significantly in sign, we calculated the fraction of positive values in the urban set and the rural set, and used the test for proportions based on normal (z) test (two-sided) (19). To determine whether the urban set and rural set differed significantly in magnitude, we took the absolute values of all the elements in the urban set and the rural set, and used the Mann-Whitney U test (two-sided) between the two sets (18). For display purposes, we used the median p-values over all three meteorological datasets in each city (main text Fig. 1, Fig. S3). We also displayed the median of (1) the medians of the urban and rural sets (main text Fig. 1), (2) the difference between the fractions of positive values in the urban set and the rural set (Fig. S3, $\Delta$Fraction positive), and (3) the difference between the medians of the absolute values of the elements in the urban set and in the rural set (Fig. S3, $\Delta$Absolute value), over all three meteorological datasets for each season and each city. Additionally, for each meteorological dataset, we calculated the Spearman correlations between the medians of the urban and rural sets and the corresponding p-values (two-sided, t-test), and displayed the medians over all three meteorological datasets to show the degree of agreement between urban and rural resilience (main text Fig. 1). The relationship between the medians of the urban and rural sets were in fact strongly linear (R^2^ $\geq$ 0.82 for all the seasons and both resistance and recovery), but neither the statistical distributions of the urban and rural sets nor the distributions of the residuals of linear regression satisfied the normality assumptions.

To determine whether the urban and rural resilience metrics differed significantly in sign (or magnitude) over the whole U.S., we calculated the fraction of positive values (or median of absolute values) over all the pixels in the urban area and all the pixels in the rural area for each meteorological dataset, city, and event. We then calculated the difference between the urban fraction of positive values (or median of absolute values) and the rural fraction (or median) for each meteorological dataset, city, and event, and used the Wilcoxon signed rank test (two-sided) to determine whether the set of differences over all the cities and events in the season were significantly different from zero for each meteorological dataset and season. Note the Wilcoxon signed rank test was appropriate here because the urban and rural samples were matched – for each meteorological dataset, event, and city, there is one urban-rural pair of fractions of positive values and one urban-rural pair of medians of absolute values) (18). We took the median p-value over all three meteorological datasets for display purpose (main text Fig. 2ab). We also displayed, for each season, the set of differences over all the cities, events in the season, and meteorological datasets as boxplots, and the medians of this set of differences in main text Fig. 2ab.

Since there were many more positive resistances than negative resistances (Fig. S2), there is the danger that a significant difference in the magnitude of the positive resistances can mask any lack of significant difference in the magnitude of the negative resistances, per the comparison in main text Fig. 2b. We verified that this is not the case at the whole U.S. level by calculating and testing the statistical significance of the differences separately for the positive and negative resilience metrics. That is, for each meteorological dataset, city, and event, we calculated the median of absolute values using only the urban and rural pixels that had positive/negative resilience metrics, and proceeded with the rest of the comparison in the same way as shown in main text Fig. 2b. The results are shown in main text Fig. 2cd.

We also examined regional variations in the urban-rural differences by determining whether the urban and rural resilience metrics differed significantly in sign and magnitude for subsets of cities. One way to obtain the subsets were to divide the 85 cities into a group in the humid eastern U.S. (to the east of the Rocky Mountains) and the arid western U.S. (to the west of the Rocky Mountains) (Table S3). The other way that we used to obtain the subsets were to divide the 85 cities based on the dominant land cover type in the rural areas of the cities (Fig. S5). We only used the rural area’s land cover to investigate the effect of land cover on the urban-rural differences in resilience because the dominant land cover type in the urban areas were always “Developed” (Fig. S5a). The dominant rural land cover of a city was the vegetated land cover that occupied the highest average fraction over 2001–2019 (Fig. S5b). For both ways of defining the subsets, the procedure of comparison between the urban and rural areas was the same as the above procedure for the whole U.S., but using the subsets of cities instead of all the cities.

### City-level regression on the urban-rural differences in resilience metrics

In Sect. 1.2.3, we obtained the difference in the fraction of positive values (or median of absolute values) between the urban area and the rural area for each meteorological dataset, city, and event. We calculated a set of predictors, which are listed and described in detail in Table S4, and examined the relationships between the predictors with the differences in the fraction of positive values (“sign”) or median of absolute values (“magnitude”), for each meteorological dataset and season, using Spearman/partial Spearman correlations and Generalized Additive Models (GAMs).

We used the Spearman/partial Spearman correlations to examine the directions of the relationships between the predictors and urban-rural differences. For all the predictors other than land cover types (LUCs), we calculated the Spearman correlations separately between each predictor and the urban-rural differences in sign or magnitude (18). We examined those predictors one-by-one, instead of together in a multiple regression setting, in view of the relatively few data points (a few hundred events) at the city level. The LUCs (Crop, Deciduous Forest, Evergreen Forest, Grass, Mixed Forest, Shrub, Wetland), however, are conceptually one group and their fractional coverages add up to near 100%, barring any remaining water, and developed area fractions not excluded by the masking procedure in Sect. 1.1.4. Therefore, for each LUCs, we calculated partial Spearman correlations between its fraction in the rural areas of the cities and the urban-rural differences, conditional on all the other land cover types (20). We used the rural land cover fractions because existing datasets cannot adequately reveal vegetation types in urban areas over an area as large as the contiguous U.S. (see Fig. S5 and main text Sect. 3.5). We determined the statistical significance of the Spearman and partial Spearman correlations using two-sided t-test (18, 20).

Because the Spearman correlations cannot be directly compared to the partial Spearman correlations to reveal the relative strengths of the LUC and non-LUC predictors, we further used GAMs for this purpose. We fitted one-predictor GAMs between the urban-rural differences in sign or magnitude and each individual non-LUC predictor, and multi-predictor GAMs between the urban-rural differences and all the LUC fractions, using Eq. 3, where $y_{i}$ is the value of the predictand at data point $i$, $X_{p,i}$ is the value of predictor $p$ in data point $i$, $N=1$ for non-land cover predictors and $N=6$ for the land cover predictors, $\beta_{0}$ is the intercept, the functions $f_{p}$ are built from penalized B splines to enable modeling nonlinear relationships, and $\epsilon_{i}$ is the residual (21). functions We used R^2^ and Akaike Information Criteria (AIC) of the fitted GAMs to Indicate the strengths of the relationships. We implemented the GAMs using the pyGAM package’s LinearGAM function (22). The parameters of the spline terms were left as default (n_splines = 20, spline_order = 3, lam = 0.6). We tested varying those parameters during preliminary analysis but did not find any large improvements in performance.

|  | $y_{i}=\beta_{0}+\sum_{p=1}^{N} f_{p}\left( X_{P,i} \right)+\epsilon_{i}$ | (3) |
| --- | --- | --- |

For display purpose, we showed the medians of the Spearman/partial Spearman correlations and their p-values, and the median R^2^ and AIC of each fitted relationship over the three meteorological datasets (main text Fig. 3). Given the fewness of the data points (~300 events per season per meteorological dataset), we did not use neural network or tree-based machine learning methods because they easily become more parameter-heavy than the GAM of Eq. 3.

The data sources and calculations of some of the predictors in Table S4 need additional clarification, which we provide as follows:

The day- and nighttime urban heat island intensities (dtmax_in/post_event, dtmin_in/post_event; Table S4) were always calculated from the same meteorological dataset as the hot months.

The Standardized Precipitation Index (SPI) and vapor pressure deficit (VPD) were only from Daymet, as it is the only available source (Table S1). To calculate the SPI, we converted the daily precipitation in Daymet during from 1981–2020 to 90-day rolling averages, fitted the rolling averages of each day of the year to gamma distributions, identified the percentile of each rolling average on the gamma distribution, and converted the percentiles to z-scores (23). We then took the daily SPI values on the last day of each month to be the SPI value of that month. For the VPD, we used the Tetens equation (24) and calculated the daily saturated vapor pressure using the average of the daily maximum and minimum temperatures. We then calculated the daily VPD as the difference between the daily saturated vapor pressure and actual vapor pressure, and averaged the daily values to obtain the monthly average. These SPI and VPD were used to calculate the moisture availability during and after the hot months (spi_in/post_event, vpd_in_post_event; Table S4), and the sensitivity of EVI to moisture availability (corr_spi_diff, corr_vpd_diff; Table S4). We interpreted the sensitivity of EVI to SPI and VPD as the water stress status of the vegetation in a pixel or a city, under the rationale that vegetation would be more sensitive to SPI and VPD if these moisture factors were more limiting on its growth (25, 26). Note that the correlation between EVI and SPI should be mostly positive, so that more positive correlations mean higher sensitivity, whereas the correlation between EVI and VPD should be mostly negative, so that more negative correlations mean higher sensitivity.

For the urban-rural difference in optimal temperatures (optimal_tmax_diff, optimal_tmin_diff; Table S4), we calculated the optimal temperatures of vegetation growth for each 1km pixel using all three meteorological datasets, averaged the result over the urban and rural areas of each city, and took the difference. We followed Yin et al. (2022) to calculate the optimal temperature in each pixel, using daily maximum (tmax) or minimum (tmin) temperatures. We first identified the growing season in a pixel by selecting the months in which the EVI was above both 0.05 and 20% of the seasonal amplitude (maximum−minimum) of EVI in the same year. We excluded the pixels that had fewer than 10 such selected EVI values during 2001–2019. For the remaining pixels, we obtained all the EVI values and the corresponding monthly mean temperatures from the three meteorological datasets. Then, we concatenated the EVI-temperature pairs across all three meteorological datasets to create a two-column table. This concatenation over all three meteorological datasets was meant to increase the number of data points available for calculating the optimal temperature, given the short study period (<20 years). Using this table and 1℃ temperature bins, we found the 90^th^ percentile of EVI in each bin and plotted the 90^th^ percentiles against the bin centers to obtain a temperature-EVI curve. We applied a running mean of every three data points on the temperature-EVI curve, and finally chose the bin center that had the highest 90^th^ percentile as the optimal temperature. Note the optimal temperature is not seasonally varying.

### Pixel-level regression on the raw resilience metrics

To better understand the nonlinear and interactive effects of the predictors on urban-rural differences in resilience metrics, we fitted random forest (28) classifiers between the pixel-level predictors and the signs (labeled 1 for positive values, and 0 for negative) of the pixel-level resilience metrics, and random forest regressors between the pixel-level predictors and the magnitude (i.e., absolute value) of the pixel-level resilience metrics. The abundance of data points at the 1km pixel level means a machine learning method like random forest was applicable. For brevity, when we refer to both the random forest classifiers and the random forest regressors, we use “random forest regression” or “regression” here and elsewhere in this paper.

To remove the influences from large scale climate and biome factors, which were already identified in the city-level regressions, we divided the cities into “land cover groups”, which are cities that are in a contiguous geographical area and have the same dominant rural land cover. Five land cover groups were considered: Deciduous Forest, Evergreen Forest, Grass, Shrub, and Wetland (Fig. S5c). We did not include crop as a land cover group because the urban-rural differences in resilience in those cities may be contaminated by sowing and harvesting signals. We used land cover to define the groups because it can jointly capture large-scale climate and biome conditions. Although there may be some overfitting in using land cover, as Figure 3 suggests, the overfitting would be acceptable because the goal here was to reduce the heterogeneity of the large-scale factors within each group. We performed separate pixel-level regressions for each land cover group, season, and meteorological dataset, using all the pixels and events available in the cities. Table S5 lists the pixel-level predictors, which were calculated in the same way as the predictors in Table S4 (Sect. 1.2.4) less the final step of taking the urban-rural differences.

For each regression, we divided the data points randomly into an 80% training set and a 20% test set. We first evaluated the performance of the regressions using conventional metrics. Those were precision, recall, F1-score (29), and Brier score (30) for the random forest classifiers, and mean bias, Pearson correlation, root mean squared error, and ratio of the standard deviation of the predicted values to the actual values for the random forest regressors. The classifiers had very high precision, recall, and F1-scores (0.79–1.00), and very low Brier scores (0.05–0.14), indicating good ability to distinguish between those event-pixels that had positive resilience metrics and those that had negative resilience metrics (Table S6). The regressors had small bias (−0.002–0.008), fair and always significant correlations (0.33–0.80), and somewhat high root mean squared errors (0.14–0.32), which is on a similar magnitude as the resistances and recoveries themselves) (Table S7). The large root mean squared errors were likely because of considerable underestimations of the standard deviation of the resilience metrics (ratio 0.36–0.81). To confirm the regressions were useful for analyzing the urban-rural differences, we calculated the urban-rural differences in the fraction of positive resilience metrics and the median absolute values of resilience metrics for each city, event, and meteorological dataset, using the predicted signs and magnitudes of the resilience metrics and following the same procedure as for the observed values (Sect. 1.2.3). We then compared the predicted and the observed urban-rural differences using Pearson correlations and linear regressions over all the cities and events for each meteorological dataset. Fig. S7–S8 show the predicted and the observed urban-rural differences over all the cities, events, and meteorological datasets, and the median Pearson correlations (including p-values [two-sided, t-test]), linear regression line (using the median slope and intercept), and R^2^ over the three meteorological datasets. The Pearson correlations were 0.32–0.84 for urban-rural differences in the sign of resistance, 0.54–0.82 for the sign of recovery, 0.52–0.93 for the magnitude of resistance, and 0.60–0.96 for the magnitude of recovery, and always statistically significant, indicating that the regressions captured the main controls of urban-rural differences (Fig. S7–S8).

After fitting the random forest regressions, we used SHapley Additive exPlanations (SHAP) values (31) to quantify the relative importance of the predictors, the partial dependence relationships between predictors and the predicted resilience metrics, and the contributions of predictors to the urban-rural differences in resilience metrics. SHAP values use game theory to explain how each predictor contributes to the output of any machine learning model at each data point. A positive SHAP value means the predictor is contributing to more positive resistance or recovery, for the random forest classifiers, and larger magnitude of resistance or recovery, for the random forest regressors, at the data point. Similarly, a negative SHAP value means the predictor is contributing to more negative resistance or recovery, or smaller magnitude of resistance or recovery, at the data point. SHAP values have the advantages of being consistent and additive (31). The consistency property ensures that the SHAP value of a predictor does not vary with the level at which a predictor is placed in a decision tree, making it suitable for application on random forest regressions (31). The additive property of the SHAP values means that the total SHAP value of all the predictors at a data point sums up to the predicted value for this data point, minus a constant that is common to all the data points (31). This additive property makes it easy to summarize the SHAP values for any subset of the data points by simply averaging them.

To calculate the importance of each predictor, we used the average absolute SHAP values of the predictor over all data points in the test set. To efficiently summarize the partial dependence relationships, we used the Spearman correlation between a predictor’s actual values and the corresponding SHAP values in the test set. A positive correlation indicates that a predictor exerted positive influence on the sign or magnitude of the resilience metric as its value increases, and a negative correlation indicates negative influence. The displayed importance, Spearman correlations, and significance of the Spearman correlations (two-sided t-test) were the medians over all three meteorological datasets (Fig. S9–S10).

To efficiently quantify the contribution of a predictor to urban-rural differences in resilience metrics in a regression, we used the Spearman correlation between the impervious fractions and the SHAP values of the predictor over all the test-set data points in this regression. We treated impervious fraction as a proxy for the level of urbanization at each pixel. Therefore, a positive/negative Spearman correlation means that a predictor is having increasingly positive/negative effect on the sign or magnitude of the resilience metric as the level of urbanization increases, and thus contributing positively/negatively to the urban-rural differences in sign or in magnitude.

We implemented the random forest classifiers and regressors using the scikit-learn version 1.0.2 package (32) and calculated the SHAP values using the shap version 0.39.0 package (<https://github.com/slundberg/shap>).

# Supplementary Figures


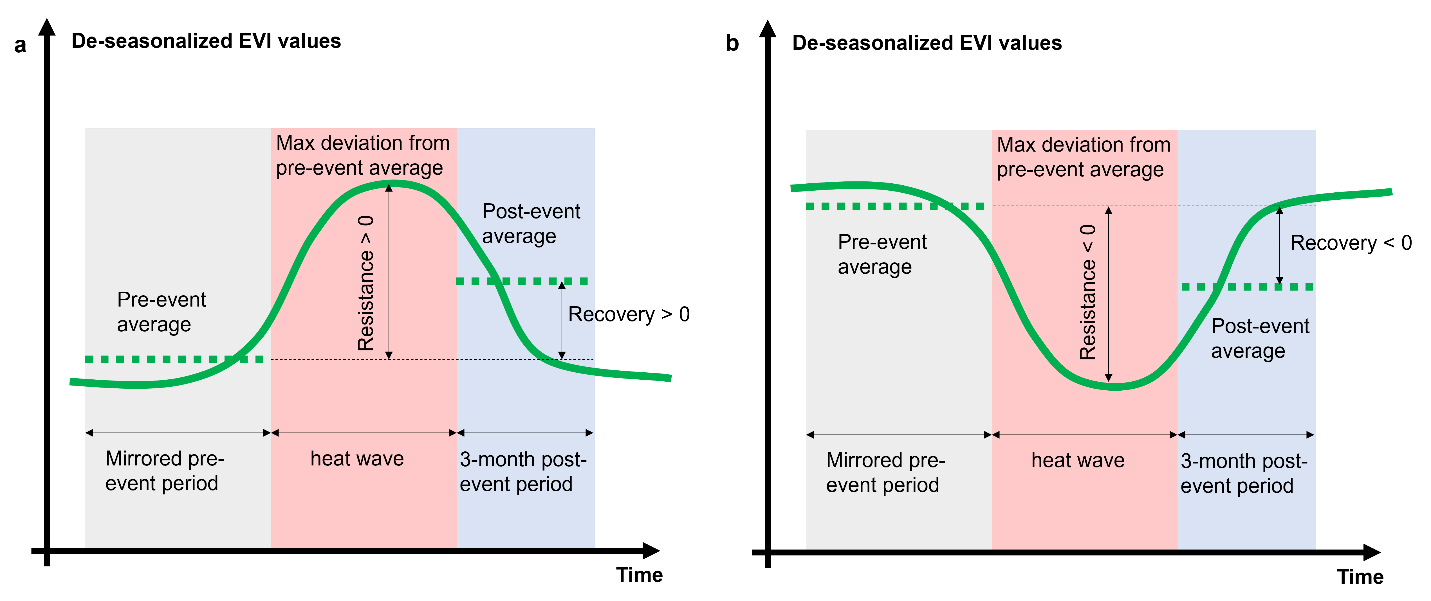


**Figure S1. Illustration of the calculation of the resistance and recovery metrics. (a) illustrates when both metrics are positive. (b) illustrates when both metrics are negative.**


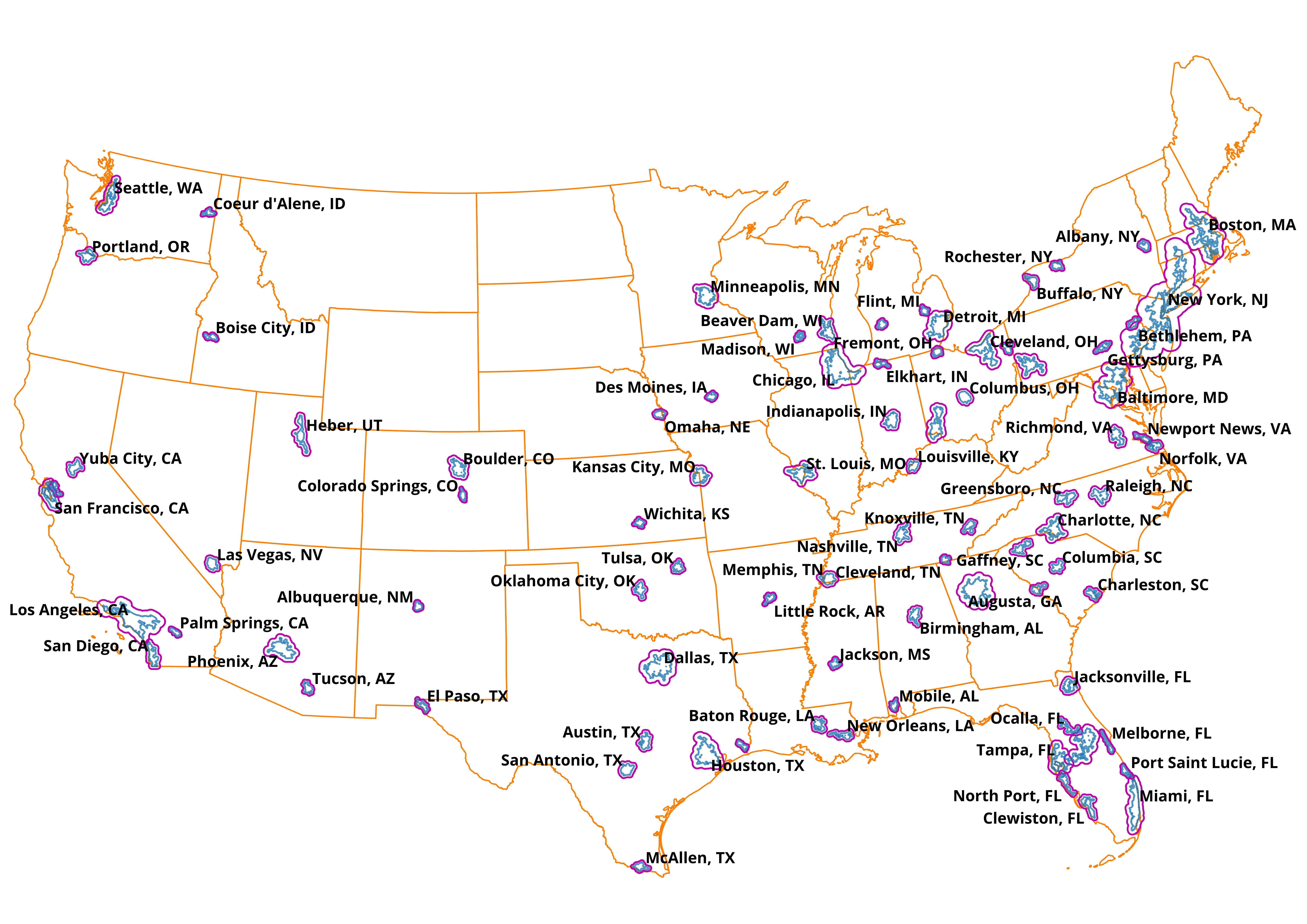


**Figure S2. Names and locations of the 85 study cities.** Blue outlines show the urban core, and purple outlines show the surrounding rural areas.


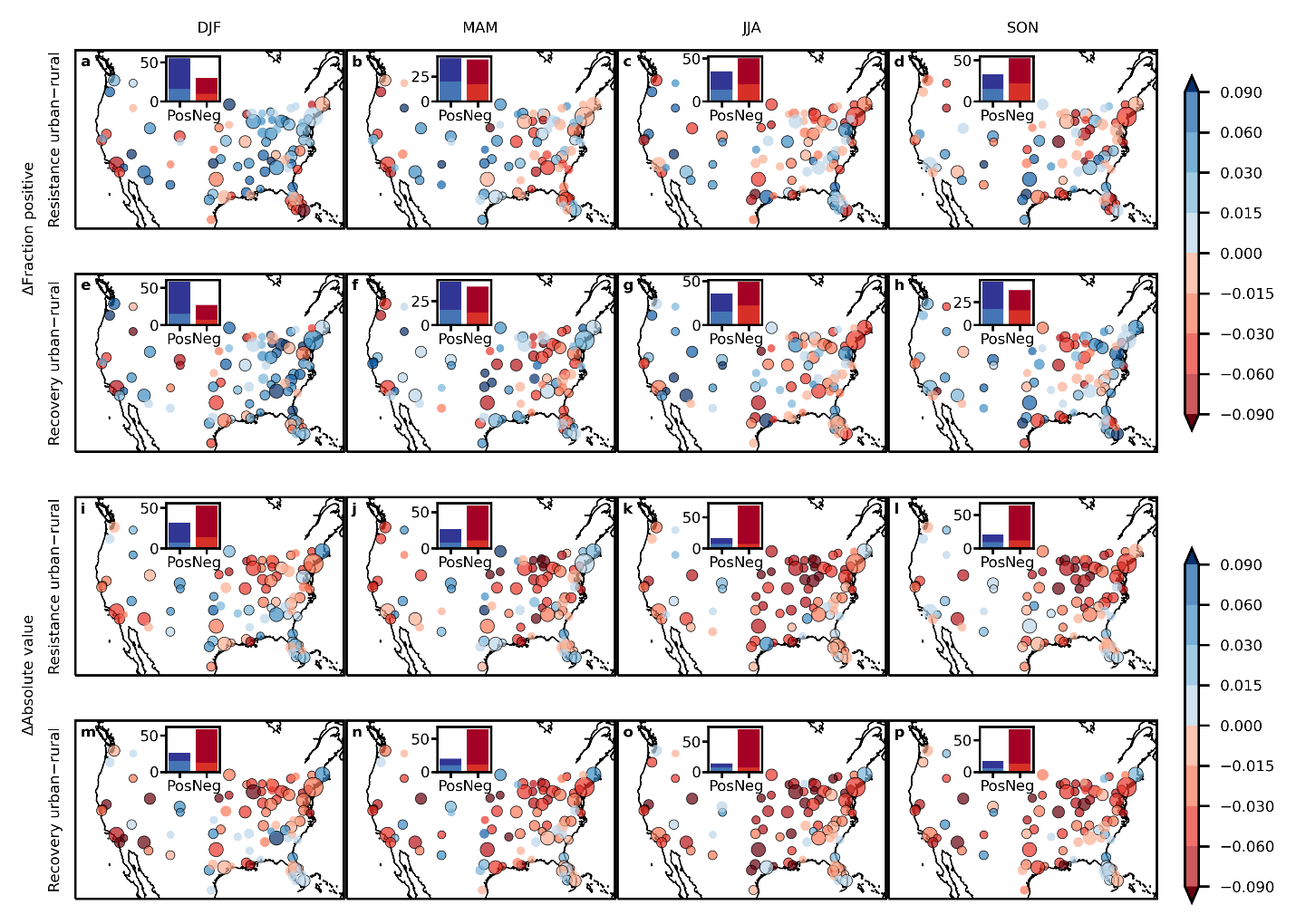


**Figure S3. Urban-rural differences in the fraction of pixels that showed positive (**$\boldsymbol{\Delta}$**Fraction positive) resistances (a–d) or recoveries (e–h), and urban-rural differences in the median magnitudes (**$\boldsymbol{\Delta}$**Absolute value) of the resistances (i–l) or recoveries (m–p).** Season abbreviations: DJF – December to February, MAM – March to May, JJA – June to August, SON – September to November. The dot sizes are proportional to the square root of city sizes. Dots with dark edges indicate that the values were significantly different from zero at p $\leq$ 0.05. The inset bars show the number of cities with positive (Pos, blue) and negative (Neg, red) median values, with darker blue/red corresponding to the number of positive/negative dots with dark edges. Exact details on the calculation of all quantities are in Sect. 1.2.3.
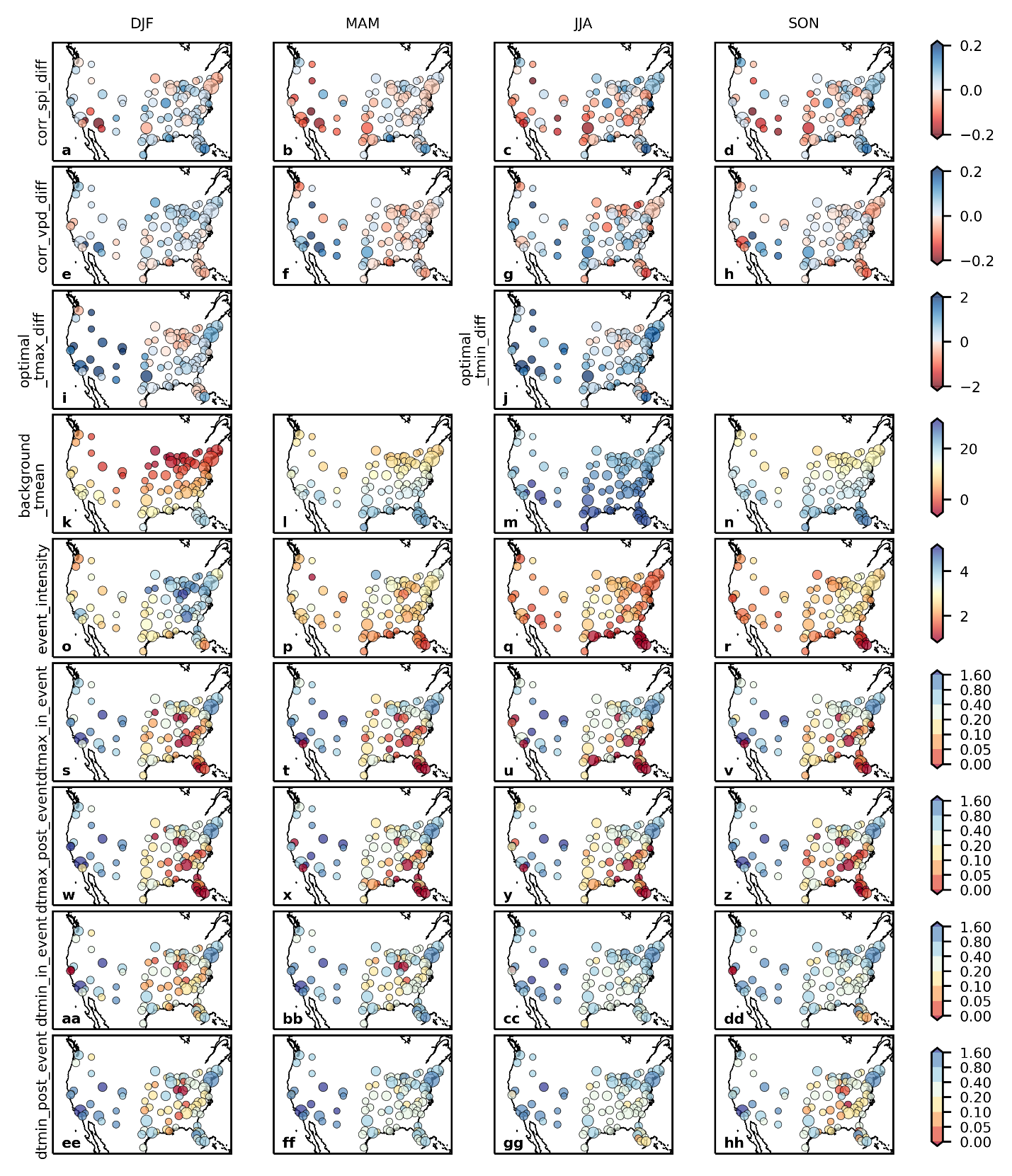


**Figure S4. Seasonal values of various predictors of urban-rural differences in the fraction of positive resistances and recoveries and the median absolute values of resistances and recoveries.** The abbreviations of the predictors are in Table S4. Note that optimal_tmax_diff and optimal_tmin_diff are the same for all the seasons of the year, hence displayed in the same row. Season abbreviations: DJF – December to February, MAM – March to May, JJA – June to August, SON – September to November. The dot sizes are proportional to the square root of city sizes.


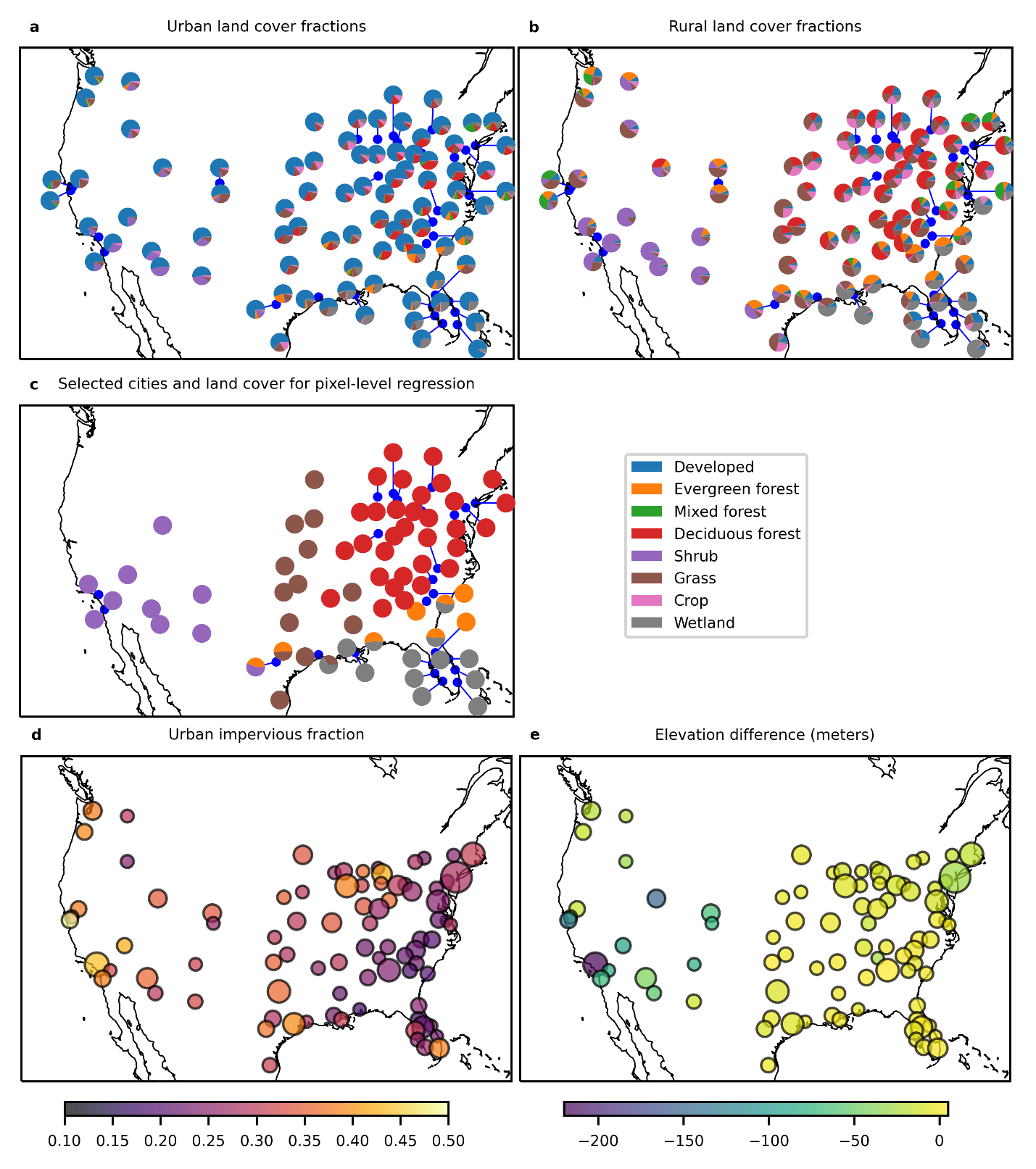


**Figure S5. Fractional land cover in the (a) urban and (b) rural areas of each city, (c) the cities included in each land cover group based on their dominant rural land covers, (d) the average impervious fraction over the urban area, and (e) the average urban-rural difference in elevation of the pixels.** The dot sizes in d and e are proportional to the square root of city sizes.


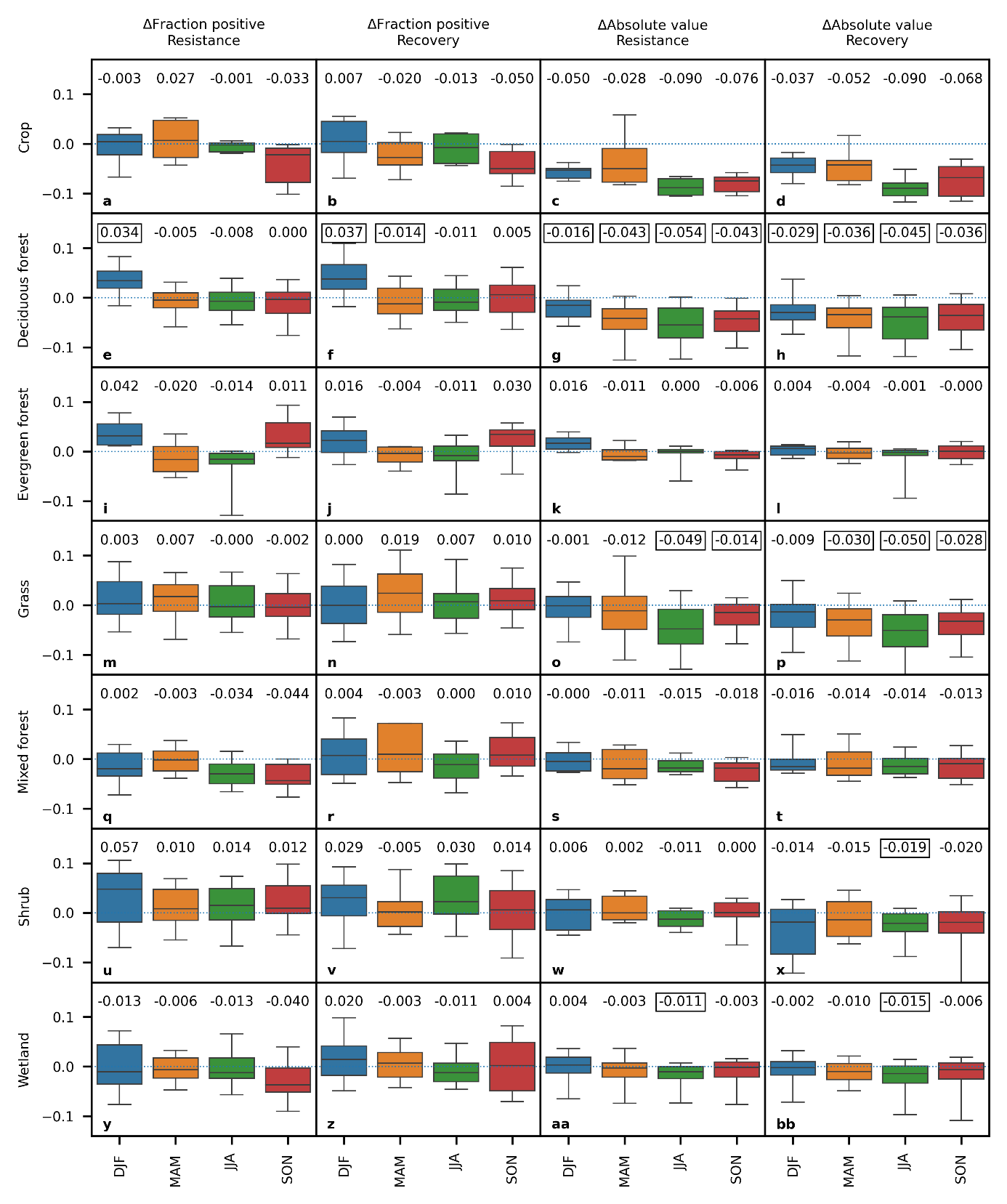


**Figure S6. Distributions of urban-rural differences in the sign (**$\boldsymbol{\Delta}$**Fraction positive) and magnitude (**$\boldsymbol{\Delta}$**Absolute value) of resilience metrics for subsets of cities that have the same dominant rural land cover types (row labels).** The boxplots show, from top to bottom, the 95^th^, 75^th^, 50^th^, 25^th^, and 5^th^ percentiles over all the hot months, cities, and meteorological data sets. Fliers are omitted for clearer view of the bulk of the distributions. The number above each boxplot shows the value of the 50^th^ percentile and whether it is significantly different from zero (bound by rectangle) at p $\leq$ 0.05. Season abbreviations: DJF – December to February, MAM – March to May, JJA – June to August, SON – September to November. Exact details of calculation of all the quantities are in Sect. 1.2.3.


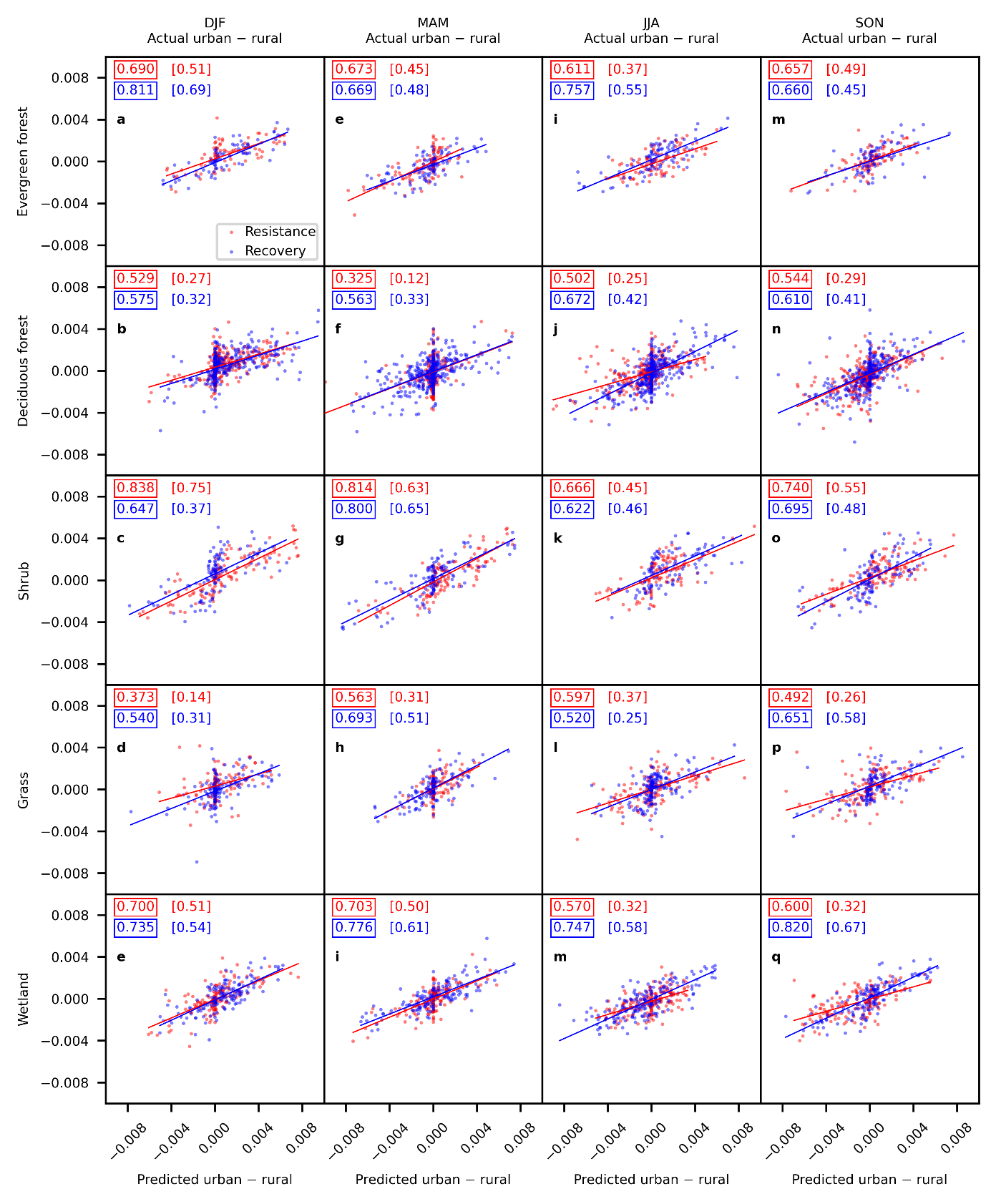


**Figure S7. Actual versus predicted urban-rural differences in the fraction of positive resistances and recoveries for cities in the same land cover groups.** The lines are fitted linear regression lines. The numbers in the left column of each panel are Pearson correlations, with bounding rectangles meaning significance at p $\leq$ 0.05. The numbers in the right column and in brackets of each panel are R^2^ values from the linear regressions. Exact details of calculation of all the quantities are in Sect. 1.2.5.


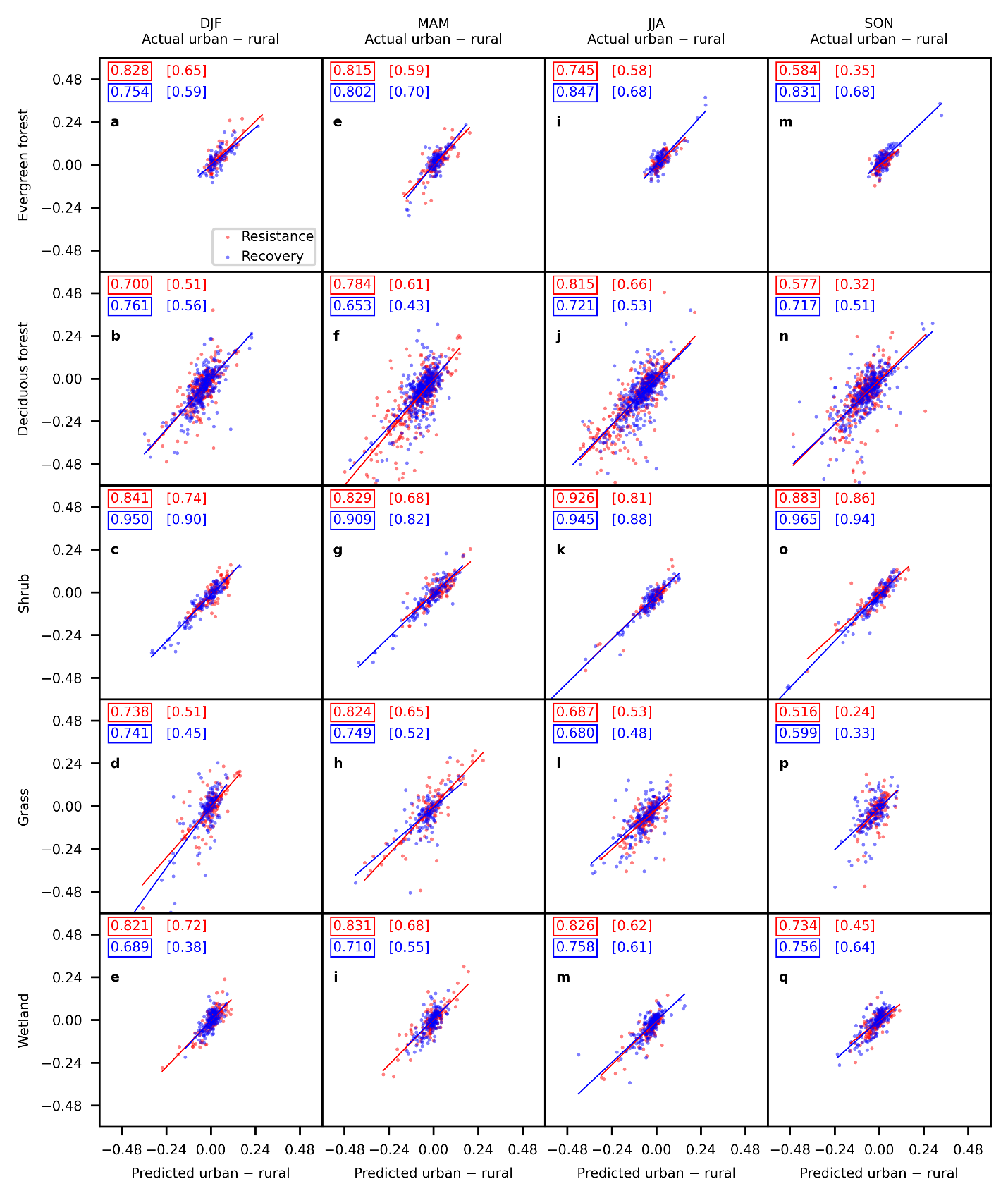


**Figure S8. Actual versus predicted urban-rural differences in the median magnitude of absolute values of resistances and recoveries for cities in the same land cover groups.** The lines are fitted linear regression lines. The numbers in the left column of each panel are Pearson correlations, with bounding rectangles meaning significance at p $\leq$ 0.05. The numbers in the right column and in brackets of each panel are R^2^ values from the linear regressions. Exact details of calculation of all the quantities are in Sect. 1.2.5.


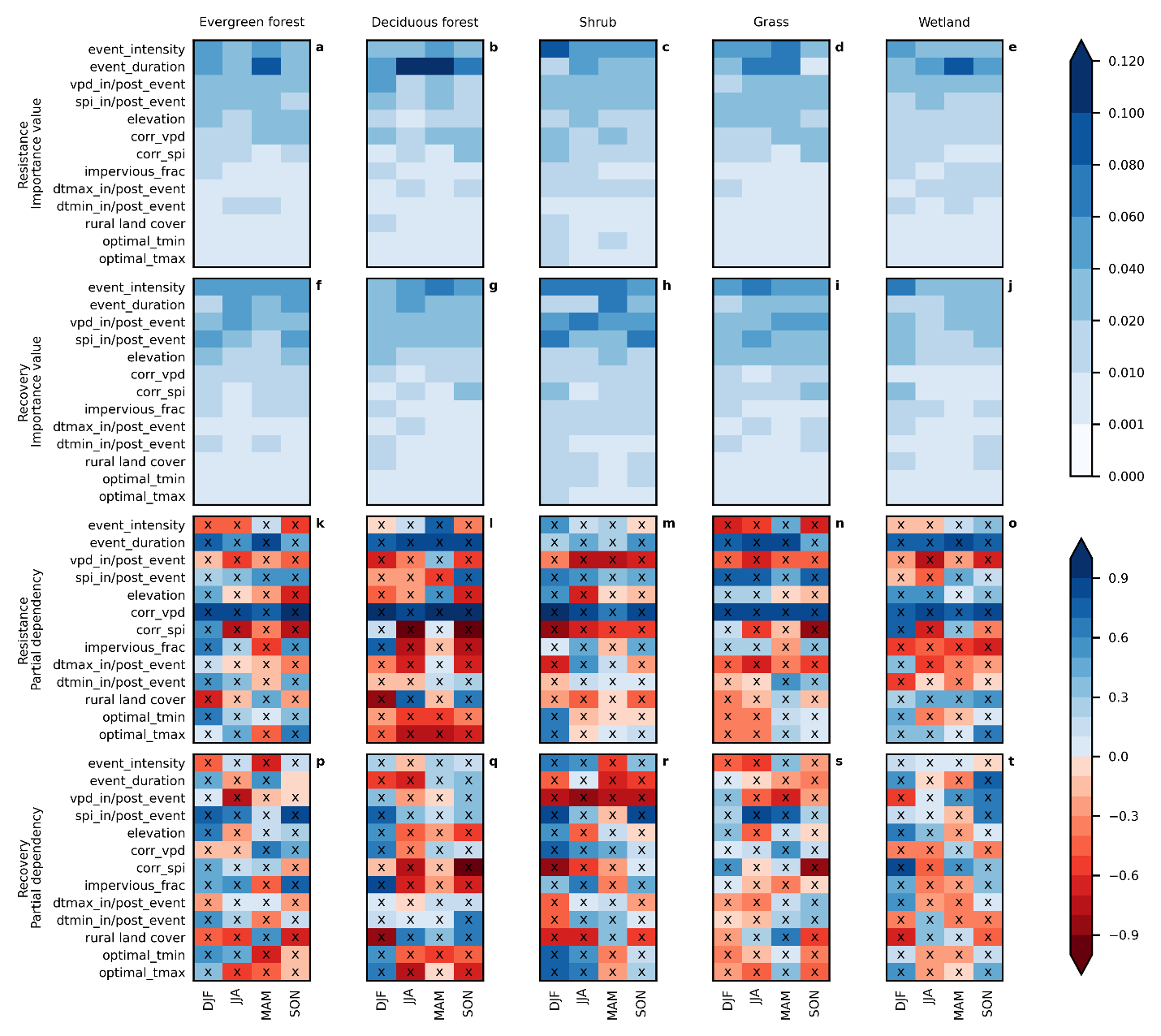


**Figure S9. (a–h) SHapley Additive exPlanations (SHAP)-value based importance of the predictors and (i–p) Spearman correlations between the SHAP values of the predictors and their actual values in the random forest classifiers**. The names of the predictors along the y-axis are explained in Table S5, and “rural land cover” refers to the dominant rural land cover of each land cover group listed along the x-axis. Non-dominant rural land cover types are omitted from the predictors. Spearman correlations are marked by “X” if they are significantly different from zero at p $\leq$ 0.05. Season abbreviations: DJF – December to February, MAM – March to May, JJA – June to August, SON – September to November. Exact details of calculation of all the quantities are in Sect. 1.2.5.


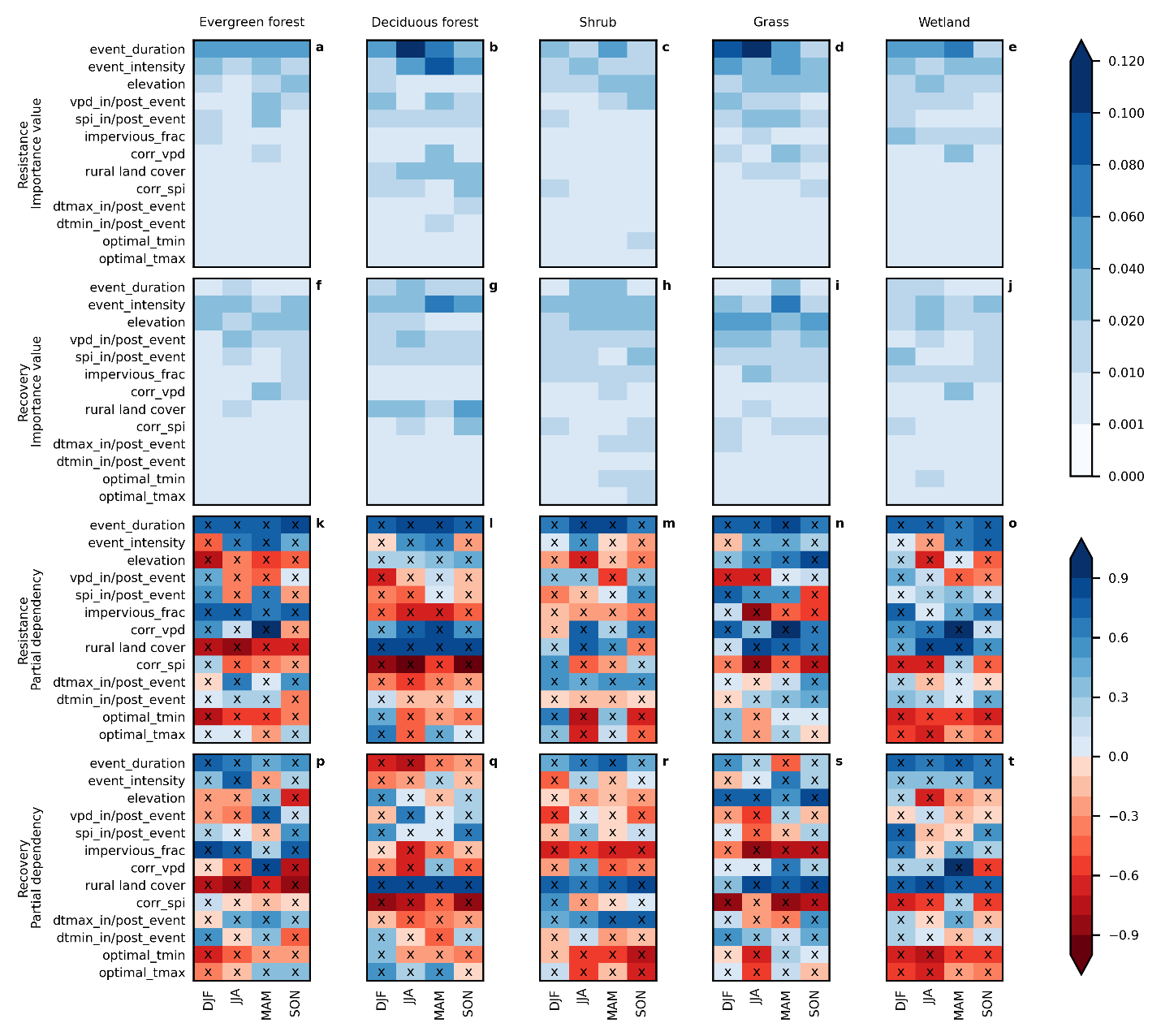


**Figure S10. (a–h) SHapley Additive exPlanations (SHAP)-value based importance of the predictors and (i–p) Spearman correlations between the SHAP values of the predictors and their actual values in the random forest regressors**. The names of the predictors along the y-axis are explained in Table S5, and “rural land cover” refers to the dominant rural land cover of each land cover group listed along the x-axis. Non-dominant rural land cover types are omitted from the predictors. Spearman correlations are marked by “X” if they are significantly different from zero at p $\leq$ 0.05. Season abbreviations: DJF – December to February, MAM – March to May, JJA – June to August, SON – September to November. Exact details of calculation of all the quantities are in Sect. 1.2.5.


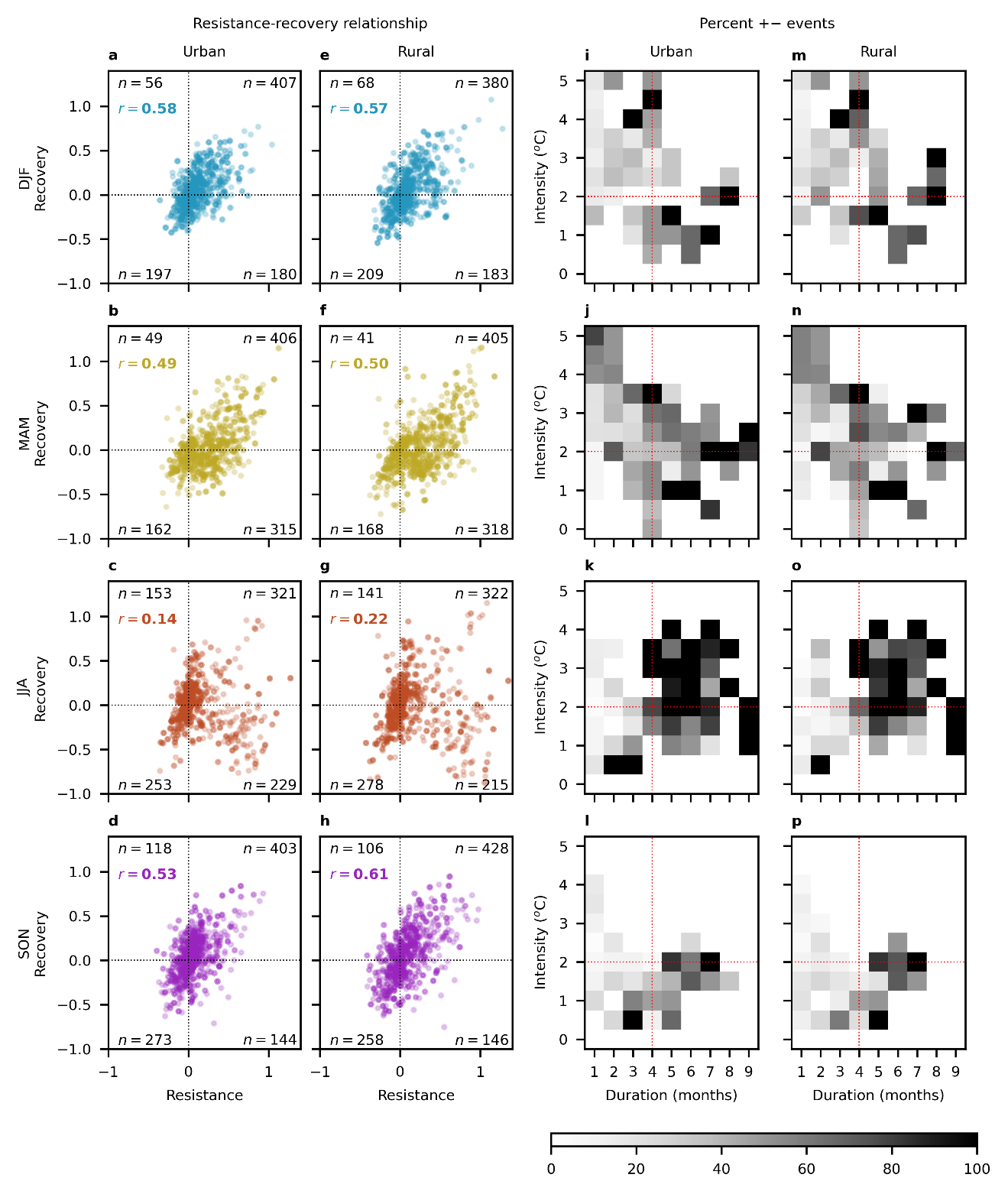


**Figure S11. Relationship between the median resistances and median recoveries of the urban (a–d) and rural (e–h) areas, and the percentages of events with positive median resistances and negative median recoveries (+−) in the urban (i–l) and rural (m–p) areas at different levels of event intensity and duration.** In a–h, each dot in the plot represents one “hot months” event in one city from one meteorological dataset. The n-values are the number of dots in each quadrant. The r-values are the Spearman correlations between the resistances and recoveries and are in bold if significantly different from zero at p ≤ 0.05 (two-sided t-test). The correlations and p-values were calculated separately for each meteorological datasets and taken the median. In i–p, the percentages were also the medians of all three meteorological datasets. Season abbreviations: DJF – December to February, MAM – March to May, JJA – June to August, SON – September to November.

# Supplementary Tables

**Table S1. Characteristics of the data sets used in this study.**

| **Name** | **Period** | **Resolution** | **Variables used here** | **Citation** |
| --- | --- | --- | --- | --- |
| Daymet v4 | 1980 – 2020 | 1 km, daily | daily maximum and minimum temperature, precipitation, vapor pressure of air | (2) |
| TOPOWx | 1948 – 2016 | 800 m, daily | daily maximum and minimum temperature | (3) |
| Zhang22 | 2003 – 2020 | 1 km, daily | daily maximum and minimum temperature | (1) |
| MOD09Q1G | 2001 – 2019 | 250 m, 8-day | Enhanced Vegetation Index (EVI) | (8) |
| NLCD | 2001, 2004, 2006, 2008, 2011, 2013, 2016, 2019 | 30 m | land cover classes, impervious fraction | (9) |
| NED | n/a | 10 m | elevation | (10) |

**Table S2. Aggregation of the National Land Cover Database (NLCD) land cover types to the land cover types used in this study.**

| **Name** | **NLCD name** | **NLCD code** |
| --- | --- | --- |
| Developed | Developed, open space | 21 |
|  | Developed, low intensity | 22 |
|  | Developed, medium intensity | 23 |
|  | Developed, high intensity | 24 |
| Deciduous forest | Deciduous forest | 41 |
| Evergreen forest | Evergreen forest | 42 |
| Mixed forest | Mixed forest | 43 |
| Shrub | Shrub/scrub | 52 |
| Grass | Grassland/herbaceous | 71 |
|  | Pasture/hay | 81 |
| Wetland | Woody wetlands | 90 |
|  | Emergent herbaceous wetlands | 95 |

**Table S3. Separately for the cities in the eastern U.S. (to the east of the Rocky Mountains) and in the western U.S. (to the west of the Rocky Mountains), urban-rural differences (“diff”) and statistical significance (p-value) in the fraction of pixels with positive resistances and recoveries (**$\boldsymbol{\Delta}$**Fraction positive), and in the median absolute resistances and recoveries over the pixels (**$\boldsymbol{\Delta}$**Absolute values).** Season abbreviations: DJF – December to February, MAM – March to May, JJA – June to August, SON – September to November. Exact details on the calculation of all quantities are in Sect. 1.2.3.

|  |  | Resistance | | | | Recovery | | | |
| --- | --- | --- | --- | --- | --- | --- | --- | --- | --- |
|  |  | Eastern | | Western | | Eastern | | Western | |
|  |  | diff | p-value | diff | p-value | diff | p-value | diff | p-value |
| $\Delta$Fraction positive | DJF | 0.024 | 0.000 | 0.012 | 0.347 | 0.023 | 0.000 | 0.008 | 0.734 |
|  | MAM | -0.002 | 0.930 | 0.011 | 0.671 | -0.006 | 0.151 | 0.019 | 0.246 |
|  | JJA | -0.012 | 0.005 | 0.024 | 0.048 | -0.010 | 0.045 | 0.031 | 0.012 |
|  | SON | -0.003 | 0.066 | 0.001 | 0.580 | 0.003 | 0.496 | 0.005 | 0.865 |
| $\Delta$Absolut value | DJF | -0.011 | 0.011 | -0.001 | 0.932 | -0.015 | 0.000 | -0.023 | 0.018 |
|  | MAM | -0.019 | 0.000 | -0.011 | 0.671 | -0.026 | 0.000 | -0.031 | 0.018 |
|  | JJA | -0.038 | 0.000 | -0.011 | 0.142 | -0.034 | 0.000 | -0.021 | 0.012 |
|  | SON | -0.032 | 0.000 | 0.000 | 0.702 | -0.025 | 0.000 | -0.020 | 0.008 |

**Table S4. Full name and description of the predictors of urban-rural differences in the sign and magnitude of resistances and recoveries.** SPI – Standardized Precipitation Index. EVI – Enhanced Vegetation Index.

| **Abbreviation** | **Meaning** | **Interpretation** | **Additional notes** |
| --- | --- | --- | --- |
| city_size_log | Logarithm of the size of the city (km^2^) | None | None |
| event_intensity | Average $\delta T_{tmax}$ during the hot months (unit: ℃). $T_{max}$ denotes the average daily maximum temperature of the whole city, and $\delta$ denotes the difference between the value during the hot months and the climatology of the value | Intensity of the hot months event | The climatology is over 2003–2019, which is the overlapping period between the three meteorological data sets |
| event_duration | Duration of the hot months (unit: months) | None | None |
| dtmax_in/post_event | Average $\Delta T_{tmax}$ during (in) or during the three months after (post) the hot months (unit: ℃). $\Delta T_{max}$ is the difference between the average daily maximum temperature of the urban pixels and the rural pixels | Daytime urban heat island intensity | “in_event” was used to predict differences in resistances and “post_event” used to predict differences in recoveries |
| dtmin_in/post_event | Average $\Delta T_{tmin}$ during (in) or during the three months after (post) the hot months (unit: ℃). $\Delta T_{min}$ is the difference average daily minimum temperature of the urban pixels and the rural pixels | Nighttime urban heat island intensity |  |
| spi_in/post_event | Average SPI of all the urban and rural pixels during (in) or during the three months after (post) the hot months | Moisture condition relative to the city’s climatology |  |
| vpd_in/post_event | Average vapor pressure deficit of all the urban and rural pixel during (in) or during the three months after (post) the hot months | Moisture condition of the city |  |
| impervious_frac | Average fraction of impervious area over all the urban pixels (unit: %) | Urban development intensity of the city | None |
| optimal_tmax_diff | Average difference in optimal daily maximum temperature for vegetation growth between the urban and the rural pixels (unit: ℃) | Daytime thermal adaptation of the urban vegetation | None |
| optimal_tmin_diff | Average difference in optimal daily minimum temperature for vegetation growth between the urban and the rural pixels (unit: ℃) | Nighttime thermal adaptation of the urban vegetation | None |
| corr_spi_diff | Average difference in the Spearman’s correlation between de-seasonalized EVI and SPI between the urban and the rural pixels, calculated separately for each season of the year | Water stress status of the vegetation | Positive (negative) corr_spi_diff is interpreted as higher (lower) water stress in the urban vegetation than rural vegetation |
| corr_vpd_diff | Average difference in the Spearman’s correlation between de-seasonalized EVI and de-seasonalized vapor pressure deficit between the urban and the rural pixels, calculated separately for each season of the year | Water stress status of the vegetation | Positive (negative) corr_vpd_diff is interpreted as lower (higher) water stress in the urban vegetation than rural vegetation |
| elev_diff | Average elevation difference between the urban and rural pixels (unit: m) | Elevation related effects (e.g., species) | None |
| background_tmean | The climatology of the average between daily maximum and minimum temperatures over all the pixels of the city during 1981-2010, calculated separately for each season of the year | Background temperature of the city | Derived from the Daymet data set only |
| background_prcp | The climatology of the average precipitation over all the pixels of the city during 1981-2010, calculated separately for each season of the year | Background precipitation of the city |  |
| Land Cover (Crop, Deciduous forest, Evergreen forest, Grass, Mixed forest, Shrub, Wetland) | Fraction of each land cover type in the rural area of the city. | None | None |

**Table S5. Full name and interpretation of the explanatory variables for pixel-level resilience and resistance.** SPI – Standardized Precipitation Index.

| **Abbreviation** | **Meaning** | **Interpretation** | **Additional notes** |
| --- | --- | --- | --- |
| event_intensity | Average $\delta T_{tmax}$ during the hot months (unit: ℃). $T_{max}$ denotes the average daily maximum temperature of the whole city, and $\delta$ denotes the difference between the value during the hot months and the climatology of the value | Intensity of the hot months | The climatology is over 2003–2019, which is the overlapping period between the three meteorological data sets |
| event_duration | Duration of the hot months (unit: months) | None | None |
| dtmax_in/post_event | Average $\Delta T_{tmax}$ during (in) or during the three months after (post) the hot months (unit: ℃). $\Delta T_{max}$ is the difference between the daily maximum temperature of the pixel and the average daily maximum temperature of the whole city | Local thermal conditions influenced by the daytime urban heat island effect | “in_event” was used to predict differences in resistances and “post_event” used to predict differences in recoveries |
| dtmin_in/post_event | Average $\Delta T_{tmin}$ during (in) or during the three months after (post) the hot months (unit: ℃). $\Delta T_{min}$ is the difference between the daily minimum temperature of the pixel and the average daily minimum temperature of the whole city | Local thermal conditions influenced by the nighttime urban heat island effect |  |
| spi_in/post_event | Average SPI of the pixels during (in) or during the three months after (post) the hot months | Moisture condition relative to the pixel’s climatology |  |
| vpd_in/post_event | Average vapor pressure deficit of the pixel during (in) or during the three months after (post) the hot months | Moisture condition of the pixel |  |
| impervious_frac | Fraction of impervious area in the pixel (unit: %) | Urbanization level of the pixel | None |
| optimal_tmax | Optimal daily maximum temperature for vegetation growth of the pixel (unit: ℃) | Daytime thermal adaptation of the vegetation | None |
| optimal_tmin | Optimal daily minimum temperature for vegetation growth of the pixel (unit: ℃) | Nighttime thermal adaptation of the vegetation | None |
| corr_spi | Spearman’s correlation between the de-seasonalized EVI and SPI of the pixel, calculated separately for each season of the year | Water stress status of the vegetation | Higher (lower) corr_spi is interpreted as higher (lower) water stress on the vegetation |
| corr_vpd | Spearman’s correlation between the de-seasonalized EVI and vapor pressure deficit of the pixel, calculated separately for each season of the year | Water stress status of the vegetation | Higher (lower) corr_vpd is interpreted as lower (higher) water stress on the vegetation |
| elev | Mean elevation of the pixel (unit: m) | Elevation related effects (e.g., species) | None |
| rural land cover | Fraction of the dominant rural land cover type of this land cover group in the pixel (unit: %). The land cover groups are Evergreen forest, Deciduous forest, Shrub, Grass, and Wetland | Fraction of the resistance or recovery signal coming from this vegetation type as opposed to impervious area or other vegetation types | None |

**Table S6. Performance statistics of the random forest classifiers on the test sets for each resilience metric, land cover group, season, and meteorological dataset.** Season abbreviations: DJF – December to February, MAM – March to May, JJA – June to August, SON – September to November.

|  |  |  | Resistance | | | | | Recovery | | | | |
| --- | --- | --- | --- | --- | --- | --- | --- | --- | --- | --- | --- | --- |
|  |  |  | Evergreen forest | Deciduous forest | Shrub | Grass | Wetland | Evergreen forest | Deciduous forest | Shrub | Grass | Wetland |
| Precision | DJF | Daymet | 0.98 | 0.87 | 0.92 | 0.90 | 0.91 | 0.97 | 0.89 | 0.98 | 0.89 | 0.95 |
|  |  | TOPOWx | 0.98 | 0.89 | 0.92 | 0.91 | 0.92 | 0.97 | 0.90 | 0.98 | 0.91 | 0.94 |
|  |  | Zhang22 | 0.99 | 0.84 | 0.93 | 0.88 | 0.90 | 0.94 | 0.89 | 0.98 | 0.90 | 0.95 |
|  | MAM | Daymet | 0.97 | 0.86 | 0.91 | 0.93 | 0.94 | 0.99 | 0.86 | 0.93 | 0.93 | 0.95 |
|  |  | TOPOWx | 0.98 | 0.88 | 0.92 | 0.91 | 0.94 | 0.97 | 0.85 | 0.93 | 0.95 | 0.95 |
|  |  | Zhang22 | 0.97 | 0.88 | 0.93 | 0.92 | 0.92 | 0.99 | 0.86 | 0.96 | 0.94 | 0.95 |
|  | JJA | Daymet | 0.98 | 0.89 | 0.94 | 0.95 | 0.92 | 0.98 | 0.83 | 0.95 | 0.91 | 0.89 |
|  |  | TOPOWx | 0.98 | 0.89 | 0.95 | 0.96 | 0.92 | 0.96 | 0.84 | 0.97 | 0.95 | 0.92 |
|  |  | Zhang22 | 0.98 | 0.92 | 0.94 | 0.94 | 0.92 | 0.98 | 0.84 | 0.95 | 0.92 | 0.88 |
|  | SON | Daymet | 0.97 | 0.86 | 0.95 | 0.95 | 0.91 | 0.97 | 0.82 | 0.98 | 0.96 | 0.94 |
|  |  | TOPOWx | 0.99 | 0.88 | 0.91 | 0.92 | 0.96 | 0.99 | 0.81 | 0.94 | 0.93 | 0.97 |
|  |  | Zhang22 | 0.98 | 0.85 | 0.94 | 0.95 | 0.92 | 0.97 | 0.82 | 0.98 | 0.93 | 0.93 |
| Recall | DJF | Daymet | 0.99 | 0.94 | 0.97 | 1.00 | 0.99 | 1.00 | 0.89 | 0.90 | 0.99 | 0.96 |
|  |  | TOPOWx | 0.99 | 0.95 | 0.95 | 0.99 | 0.99 | 1.00 | 0.87 | 0.90 | 0.98 | 0.98 |
|  |  | Zhang22 | 0.99 | 0.96 | 0.98 | 1.00 | 0.99 | 1.00 | 0.87 | 0.89 | 0.99 | 0.97 |
|  | MAM | Daymet | 0.99 | 0.98 | 0.96 | 0.98 | 0.98 | 0.96 | 0.79 | 0.92 | 0.91 | 0.95 |
|  |  | TOPOWx | 1.00 | 0.98 | 0.97 | 1.00 | 1.00 | 0.99 | 0.86 | 0.94 | 0.99 | 0.97 |
|  |  | Zhang22 | 0.99 | 0.98 | 0.95 | 0.98 | 0.99 | 0.95 | 0.84 | 0.90 | 0.92 | 0.95 |
|  | JJA | Daymet | 0.99 | 0.87 | 0.90 | 0.91 | 0.99 | 0.99 | 0.91 | 0.91 | 0.90 | 0.98 |
|  |  | TOPOWx | 0.99 | 0.96 | 0.94 | 0.97 | 0.99 | 0.97 | 0.93 | 0.92 | 0.94 | 0.98 |
|  |  | Zhang22 | 0.99 | 0.87 | 0.92 | 0.91 | 0.99 | 0.99 | 0.88 | 0.92 | 0.93 | 0.98 |
|  | SON | Daymet | 0.99 | 0.91 | 0.93 | 0.94 | 0.97 | 1.00 | 0.96 | 0.91 | 0.85 | 0.96 |
|  |  | TOPOWx | 0.98 | 0.97 | 0.94 | 0.96 | 0.95 | 0.98 | 0.97 | 0.94 | 0.94 | 0.88 |
|  |  | Zhang22 | 1.00 | 0.94 | 0.94 | 0.92 | 0.97 | 1.00 | 0.96 | 0.92 | 0.88 | 0.95 |
| F1-score | DJF | Daymet | 0.99 | 0.90 | 0.95 | 0.95 | 0.95 | 0.98 | 0.89 | 0.94 | 0.94 | 0.96 |
|  |  | TOPOWx | 0.99 | 0.92 | 0.94 | 0.95 | 0.95 | 0.98 | 0.88 | 0.94 | 0.94 | 0.96 |
|  |  | Zhang22 | 0.99 | 0.90 | 0.95 | 0.94 | 0.94 | 0.97 | 0.88 | 0.93 | 0.94 | 0.96 |
|  | MAM | Daymet | 0.98 | 0.92 | 0.93 | 0.95 | 0.96 | 0.98 | 0.83 | 0.93 | 0.92 | 0.95 |
|  |  | TOPOWx | 0.99 | 0.92 | 0.95 | 0.95 | 0.97 | 0.98 | 0.86 | 0.94 | 0.97 | 0.96 |
|  |  | Zhang22 | 0.98 | 0.92 | 0.94 | 0.95 | 0.96 | 0.97 | 0.85 | 0.93 | 0.93 | 0.95 |
|  | JJA | Daymet | 0.99 | 0.88 | 0.92 | 0.93 | 0.95 | 0.98 | 0.87 | 0.93 | 0.90 | 0.93 |
|  |  | TOPOWx | 0.98 | 0.93 | 0.94 | 0.96 | 0.96 | 0.97 | 0.89 | 0.95 | 0.94 | 0.95 |
|  |  | Zhang22 | 0.99 | 0.89 | 0.93 | 0.93 | 0.95 | 0.99 | 0.86 | 0.94 | 0.93 | 0.93 |
|  | SON | Daymet | 0.98 | 0.88 | 0.94 | 0.94 | 0.94 | 0.98 | 0.89 | 0.94 | 0.90 | 0.95 |
|  |  | TOPOWx | 0.99 | 0.92 | 0.93 | 0.94 | 0.96 | 0.98 | 0.88 | 0.94 | 0.93 | 0.92 |
|  |  | Zhang22 | 0.99 | 0.89 | 0.94 | 0.94 | 0.94 | 0.99 | 0.88 | 0.95 | 0.90 | 0.94 |
| Brier score | DJF | Daymet | 0.068 | 0.122 | 0.077 | 0.078 | 0.094 | 0.060 | 0.119 | 0.064 | 0.096 | 0.084 |
|  |  | TOPOWx | 0.066 | 0.113 | 0.081 | 0.086 | 0.098 | 0.065 | 0.125 | 0.062 | 0.093 | 0.093 |
|  |  | Zhang22 | 0.066 | 0.118 | 0.078 | 0.092 | 0.092 | 0.068 | 0.117 | 0.067 | 0.096 | 0.098 |
|  | MAM | Daymet | 0.056 | 0.091 | 0.095 | 0.074 | 0.089 | 0.072 | 0.141 | 0.081 | 0.099 | 0.114 |
|  |  | TOPOWx | 0.054 | 0.082 | 0.094 | 0.068 | 0.086 | 0.073 | 0.130 | 0.079 | 0.080 | 0.107 |
|  |  | Zhang22 | 0.062 | 0.084 | 0.089 | 0.067 | 0.094 | 0.077 | 0.138 | 0.077 | 0.089 | 0.115 |
|  | JJA | Daymet | 0.079 | 0.126 | 0.099 | 0.100 | 0.112 | 0.079 | 0.128 | 0.084 | 0.098 | 0.131 |
|  |  | TOPOWx | 0.077 | 0.116 | 0.088 | 0.074 | 0.094 | 0.092 | 0.118 | 0.073 | 0.087 | 0.119 |
|  |  | Zhang22 | 0.075 | 0.121 | 0.106 | 0.098 | 0.116 | 0.075 | 0.134 | 0.085 | 0.089 | 0.131 |
|  | SON | Daymet | 0.065 | 0.132 | 0.090 | 0.106 | 0.113 | 0.064 | 0.131 | 0.067 | 0.096 | 0.095 |
|  |  | TOPOWx | 0.088 | 0.118 | 0.118 | 0.110 | 0.108 | 0.065 | 0.127 | 0.074 | 0.096 | 0.109 |
|  |  | Zhang22 | 0.064 | 0.135 | 0.102 | 0.107 | 0.113 | 0.057 | 0.132 | 0.073 | 0.100 | 0.098 |

**Table S7. Performance statistics of the random forest regressors on the test sets for each resilience metric, land cover group, season, and meteorological dataset.** Abbreviations: bias – mean bias, corr – Pearson correlation coefficient (bold means significantly different from zero at p ≤ 0.05 according to two-sided t-tests), rmse – root mean squared error, std_ratio – the ratio of the standard deviation of the predicted values to the standard deviation of the actual values. Season abbreviations: DJF – December to February, MAM – March to May, JJA – June to August, SON – September to November.

|  |  |  | Resistance | | | | | Recovery | | | | |
| --- | --- | --- | --- | --- | --- | --- | --- | --- | --- | --- | --- | --- |
|  |  |  | Evergreen forest | Deciduous forest | Shrub | Grass | Wetland | Evergreen forest | Deciduous forest | Shrub | Grass | Wetland |
| bias | DJF | Daymet | 0.006 | 0.002 | 0.000 | -0.001 | 0.003 | 0.004 | -0.002 | 0.001 | 0.003 | 0.001 |
|  |  | TOPOWx | 0.003 | 0.003 | 0.001 | 0.002 | 0.001 | 0.005 | 0.003 | 0.000 | -0.002 | 0.001 |
|  |  | Zhang22 | 0.004 | 0.004 | 0.002 | 0.000 | -0.001 | 0.003 | 0.003 | 0.001 | 0.000 | -0.001 |
|  | MAM | Daymet | 0.004 | -0.002 | 0.003 | 0.004 | 0.003 | 0.007 | 0.000 | 0.002 | 0.003 | 0.001 |
|  |  | TOPOWx | 0.000 | -0.002 | 0.001 | 0.000 | 0.003 | -0.001 | 0.000 | 0.001 | -0.002 | 0.001 |
|  |  | Zhang22 | 0.008 | 0.000 | 0.003 | 0.002 | 0.001 | 0.004 | 0.001 | 0.002 | 0.000 | -0.001 |
|  | JJA | Daymet | 0.006 | 0.001 | 0.003 | 0.001 | 0.002 | 0.007 | 0.001 | 0.003 | 0.001 | 0.002 |
|  |  | TOPOWx | 0.003 | 0.002 | 0.002 | 0.002 | 0.003 | 0.001 | 0.002 | 0.002 | 0.003 | 0.005 |
|  |  | Zhang22 | 0.000 | 0.001 | 0.002 | 0.005 | 0.002 | 0.003 | -0.001 | 0.001 | 0.004 | 0.002 |
|  | SON | Daymet | 0.007 | 0.002 | 0.001 | 0.004 | 0.004 | -0.001 | -0.001 | 0.001 | 0.005 | 0.004 |
|  |  | TOPOWx | 0.004 | 0.001 | 0.001 | 0.002 | 0.002 | 0.004 | 0.001 | 0.000 | 0.000 | 0.001 |
|  |  | Zhang22 | 0.005 | 0.003 | 0.000 | 0.005 | 0.004 | 0.005 | 0.003 | -0.001 | 0.004 | 0.002 |
| corr | DJF | Daymet | **0.493** | **0.392** | **0.571** | **0.590** | **0.484** | **0.386** | **0.367** | **0.612** | **0.425** | **0.448** |
|  |  | TOPOWx | **0.420** | **0.400** | **0.512** | **0.646** | **0.461** | **0.369** | **0.362** | **0.610** | **0.471** | **0.473** |
|  |  | Zhang22 | **0.491** | **0.438** | **0.500** | **0.562** | **0.510** | **0.335** | **0.401** | **0.601** | **0.439** | **0.494** |
|  | MAM | Daymet | **0.486** | **0.571** | **0.563** | **0.695** | **0.521** | **0.387** | **0.435** | **0.626** | **0.575** | **0.380** |
|  |  | TOPOWx | **0.604** | **0.587** | **0.538** | **0.633** | **0.541** | **0.428** | **0.432** | **0.594** | **0.504** | **0.400** |
|  |  | Zhang22 | **0.474** | **0.590** | **0.594** | **0.620** | **0.533** | **0.359** | **0.447** | **0.629** | **0.554** | **0.409** |
|  | JJA | Daymet | **0.477** | **0.668** | **0.538** | **0.737** | **0.565** | **0.501** | **0.448** | **0.603** | **0.522** | **0.511** |
|  |  | TOPOWx | **0.417** | **0.690** | **0.511** | **0.804** | **0.592** | **0.465** | **0.470** | **0.610** | **0.447** | **0.505** |
|  |  | Zhang22 | **0.434** | **0.688** | **0.636** | **0.742** | **0.536** | **0.483** | **0.422** | **0.703** | **0.500** | **0.503** |
|  | SON | Daymet | **0.336** | **0.468** | **0.572** | **0.419** | **0.408** | **0.3**  **35** | **0.435** | **0.669** | **0.453** | **0.441** |
|  |  | TOPOWx | **0.366** | **0.465** | **0.538** | **0.415** | **0.479** | **0.400** | **0.404** | **0.678** | **0.420** | **0.431** |
|  |  | Zhang22 | **0.352** | **0.465** | **0.637** | **0.396** | **0.394** | **0.403** | **0.422** | **0.730** | **0.463** | **0.421** |
| rmse | DJF | Daymet | 0.170 | 0.269 | 0.162 | 0.237 | 0.194 | 0.187 | 0.278 | 0.175 | 0.248 | 0.204 |
|  |  | TOPOWx | 0.154 | 0.270 | 0.145 | 0.232 | 0.186 | 0.188 | 0.282 | 0.165 | 0.247 | 0.203 |
|  |  | Zhang22 | 0.169 | 0.273 | 0.154 | 0.217 | 0.203 | 0.190 | 0.279 | 0.173 | 0.240 | 0.205 |
|  | MAM | Daymet | 0.192 | 0.284 | 0.152 | 0.245 | 0.198 | 0.192 | 0.269 | 0.174 | 0.252 | 0.208 |
|  |  | TOPOWx | 0.201 | 0.291 | 0.149 | 0.253 | 0.194 | 0.206 | 0.275 | 0.176 | 0.253 | 0.202 |
|  |  | Zhang22 | 0.188 | 0.290 | 0.158 | 0.240 | 0.198 | 0.189 | 0.276 | 0.174 | 0.237 | 0.206 |
|  | JJA | Daymet | 0.141 | 0.244 | 0.143 | 0.202 | 0.158 | 0.179 | 0.298 | 0.165 | 0.244 | 0.191 |
|  |  | TOPOWx | 0.151 | 0.244 | 0.141 | 0.211 | 0.166 | 0.183 | 0.298 | 0.162 | 0.239 | 0.190 |
|  |  | Zhang22 | 0.144 | 0.242 | 0.145 | 0.202 | 0.156 | 0.188 | 0.287 | 0.172 | 0.243 | 0.195 |
|  | SON | Daymet | 0.168 | 0.278 | 0.147 | 0.188 | 0.172 | 0.206 | 0.326 | 0.173 | 0.238 | 0.205 |
|  |  | TOPOWx | 0.160 | 0.274 | 0.160 | 0.201 | 0.172 | 0.193 | 0.312 | 0.191 | 0.255 | 0.193 |
|  |  | Zhang22 | 0.160 | 0.277 | 0.158 | 0.190 | 0.165 | 0.210 | 0.320 | 0.186 | 0.239 | 0.207 |
| std_ratio | DJF | Daymet | 0.544 | 0.399 | 0.572 | 0.596 | 0.510 | 0.441 | 0.370 | 0.590 | 0.440 | 0.448 |
|  |  | TOPOWx | 0.466 | 0.407 | 0.498 | 0.657 | 0.457 | 0.406 | 0.365 | 0.597 | 0.499 | 0.451 |
|  |  | Zhang22 | 0.533 | 0.447 | 0.515 | 0.574 | 0.496 | 0.399 | 0.403 | 0.582 | 0.438 | 0.456 |
|  | MAM | Daymet | 0.551 | 0.565 | 0.575 | 0.694 | 0.557 | 0.450 | 0.446 | 0.613 | 0.580 | 0.375 |
|  |  | TOPOWx | 0.630 | 0.582 | 0.541 | 0.653 | 0.554 | 0.448 | 0.433 | 0.582 | 0.525 | 0.377 |
|  |  | Zhang22 | 0.538 | 0.583 | 0.614 | 0.628 | 0.538 | 0.418 | 0.441 | 0.625 | 0.566 | 0.376 |
|  | JJA | Daymet | 0.533 | 0.667 | 0.545 | 0.746 | 0.572 | 0.551 | 0.452 | 0.594 | 0.527 | 0.521 |
|  |  | TOPOWx | 0.468 | 0.687 | 0.513 | 0.810 | 0.606 | 0.509 | 0.466 | 0.586 | 0.464 | 0.503 |
|  |  | Zhang22 | 0.480 | 0.692 | 0.641 | 0.760 | 0.538 | 0.523 | 0.411 | 0.695 | 0.507 | 0.482 |
|  | SON | Daymet | 0.397 | 0.467 | 0.566 | 0.461 | 0.420 | 0.394 | 0.418 | 0.658 | 0.481 | 0.439 |
|  |  | TOPOWx | 0.443 | 0.459 | 0.536 | 0.457 | 0.508 | 0.458 | 0.400 | 0.641 | 0.420 | 0.448 |
|  |  | Zhang22 | 0.448 | 0.464 | 0.626 | 0.426 | 0.429 | 0.461 | 0.427 | 0.714 | 0.468 | 0.430 |

# References

1. T. Zhang, *et al.*, A global dataset of daily maximum and minimum near-surface air temperature at 1 km resolution over land (2003–2020). *Earth Syst. Sci. Data* **14**, 5637–5649 (2022).

2. P. E. Thornton, *et al.*, Gridded daily weather data for North America with comprehensive uncertainty quantification. *Sci. Data* **8**, 190 (2021).

3. J. W. Oyler, A. Ballantyne, K. Jencso, M. Sweet, S. W. Running, Creating a topoclimatic daily air temperature dataset for the conterminous United States using homogenized station data and remotely sensed land skin temperature. *Int. J. Climatol* **35**, 2258–2279 (2015).

4. Y. Zeng, *et al.*, Optical vegetation indices for monitoring terrestrial ecosystems globally. *Nat. Rev. Earth Environ.* **3**, 477–493 (2022).

5. S. Wang, *et al.*, Urban−rural gradients reveal joint control of elevated CO_2_ and temperature on extended photosynthetic seasons. *Nat. Ecol. Evol.* **3**, 1076–1085 (2019).

6. L. Zhang, *et al.*, Direct and indirect impacts of urbanization on vegetation growth across the world’s cities. *Sci. Adv.* **8**, eabo0095 (2022).

7. LAADS DAAC, MODIS for North American Carbon Program (2022) (November 23, 2022).

8. F. Gao, *et al.*, An algorithm to produce temporally and spatially continuous MODIS-LAI time series. *IEEE Geosci. Remote Sensing Lett.* **5**, 60–64 (2008).

9. J. Dewitz, U.S. Geological Survey, National Land Cover Database (NLCD) 2019 Products (ver. 2.0, June 2021): U.S. Geological Survey data release (2021) https:/doi.org/10.5066/P9KZCM54 (December 2, 2022).

10. D. B. Gesch, G. A. Evans, M. J. Oimoen, S. Arundel, “The National Elevation Dataset” in (American Society for Photogrammetry and Remote Sensing, 2018), pp. 83–110.

11. Y. Zhou, *et al.*, A global map of urban extent from nightlights. *Environ. Res. Lett.* **10**, 054011 (2015).

12. L. Meng, *et al.*, Urban warming advances spring phenology but reduces the response of phenology to temperature in the conterminous United States. *Proc. Natl. Acad. Sci. USA* **117**, 4228–4233 (2020).

13. Y. Yao, *et al.*, Evaluation of ecosystem resilience to drought based on drought intensity and recovery time. *Agric. For. Meteorol.* **314**, 108809 (2022).

14. T. Ermitão, C. M. Gouveia, A. Bastos, A. C. Russo, Vegetation productivity losses linked to Mediterranean hot and dry events. *Remote Sens.* **13**, 4010 (2021).

15. F. Isbell, *et al.*, Biodiversity increases the resistance of ecosystem productivity to climate extremes. *Nat.* **526**, 574–577 (2015).

16. T. Zheng, Disentangling biology from mathematical necessity in twentieth-century gymnosperm resilience trends. *Nat. Ecol. Evol.* **5**, 733–735 (2021).

17. C. R. Schwalm, *et al.*, Global patterns of drought recovery. *Nature* **548**, 202–205 (2017).

18. M. Hollander, D. A. Wolfe, E. Chicken, *Nonparametric statistical methods*, 3rd Ed. (Wiley, 2013).

19. A. Agresti, *An introduction to categorical data analysis*, 3rd Ed. (John Wiley & Sons, 2018).

20. S. Kim, ppcor: An R Package for a Fast Calculation to Semi-partial Correlation Coefficients. *Commun Stat Appl Methods* **22**, 665–674 (2015).

21. G. James, D. Witten, T. Hastie, R. Tibshirani, J. Taylor, “Moving Beyond Linearity” in *An Introduction to Statistical Learning: With Applications in Python*, Springer Texts in Statistics., G. James, D. Witten, T. Hastie, R. Tibshirani, J. Taylor, Eds. (Springer International Publishing, 2023), pp. 289–329.

22. , dswah/pyGAM: v0.8.0 (October 15, 2023).

23. T. B. McKee, N. J. Doesken, J. Kleist, The relationship of drought frequency and duration to time scales in (1993), pp. 1–6.

24. J. L. Monteith, M. H. Unsworth, “Chapter 2 Properties of Gases and Liquids” in *Principles of Environmental Physics (Fourth Edition)*, (AP, Amsterdam, 2008), pp. 5–23.

25. G. E. Maurer, A. J. Hallmark, R. F. Brown, O. E. Sala, S. L. Collins, Sensitivity of primary production to precipitation across the United States. *Ecol. Lett.* **23**, 527–536 (2020).

26. Q. Zhang, *et al.*, Response of ecosystem intrinsic water use efficiency and gross primary productivity to rising vapor pressure deficit. *Environ. Res. Lett.* **14**, 074023 (2019).

27. G. Yin, A. Verger, A. Descals, I. Filella, J. Peñuelas, Nonlinear thermal responses outweigh water limitation in the attenuated effect of climatic warming on photosynthesis in northern ecosystems. *Geophys. Res. Lett.* **49** (2022).

28. L. Breiman, Random Forests. *Machine Learning* **45**, 5–32 (2001).

29. T. Fawcett, An introduction to ROC analysis. *Pattern Recognit. Lett.* **27**, 861–874 (2006).

30. G. W. Brier, Verification of forecasts expressed in terms of probability. *Mon. Weather Rev.* **78**, 1–3 (1950).

31. S. M. Lundberg, S.-I. Lee, “A unified approach to interpreting model predictions” in *Advances in Neural Information Processing Systems 30*, I. Guyon, *et al.*, Eds. (Curran Associates, Inc., 2017), pp. 4765–4774.

32. F. Pedregosa, *et al.*, Scikit-learn: machine learning in Python. *J. Mach. Learn. Res.* **12**, 2825–2830 (2011).
